# Strontium in public drinking water and associated public health risks in Chinese cities

**DOI:** 10.1007/s11356-021-12378-y

**Published:** 2021-01-12

**Authors:** Hao Peng, Feifei Yao, Shuang Xiong, Zhonghua Wu, Geng Niu, Taotao Lu

**Affiliations:** 1 School of Environmental Studies, China University of Geoscience, Wuhan, 430078 China; 2 Qingdao Haier Smart Technology R&D Co., Ltd, Qingdao, 266101 China; 3 Wuhan Zondy W&R Environmental Technology Co., Ltd, Wuhan, 430078 China; 4 Department of Hydrology, University of Bayreuth, 95440 Bayreuth, Germany

**Keywords:** Public drinking water, Strontium, Daily intake, Health risk

## Abstract

Due to the fact that strontium (Sr) is not involved in the scope of supervision of drinking water in China, the Sr concentration in public drinking water and its related health risks have been neglected for a long time. In this research, public drinking water samples were collected from 314 cities across the country to reveal the concentration and spatial distribution of Sr in public drinking water. In addition, the Monte Carlo method (a statistical simulation method) was applied to evaluate the Sr intake from drinking water and human health risks among different age groups and different regions. As shown in the results, the Sr was in the concentration range of 0.005–3.11 mg/L with a mean value of 0.360 mg/L. There were significant differences in the Sr concentration in different regions; in general, it was high in the north and low in the south. The Sr intakes of infants, children, teens, and adults from drinking water were 0.273, 0.503, 0.633, and 0.784 mg/day, respectively. There was a significant positive correlation between Sr concentration in drinking water and bone mineral density (BMD) in the elderly. Especially, the correlation coefficients (r) between Sr concentration and the BMD of the elderly whose age fell in the range of 60–70 years were 0.692 (male) and 0.483 (female). In addition, the Sr concentration in drinking water was positively correlated with the incidence of children’s rickets (*r* = 0.411), while the Ca/Br ratio was negatively correlated with the incidence of children’s rickets (*r* = − 0.410). According to the health risk assessment, among people of different ages, infants’ hazard index (HI) value was the highest. The mean value and 95th percentile value were 0.066 and 0.247. Non-carcinogenic risk of Sr through drinking water among different people in different regions was less than 1, which meant no significant damage to human health. This study is the first time to systematically investigate Sr in public drinking water across the whole country. More importantly, the conclusions can be applied to risk control and management of public drinking water.

**Supplementary Information:**

The online version contains supplementary material available at 10.1007/s11356-021-12378-y.

## Introduction

Strontium (Sr), which accounts for 0.02–0.03% of the earth’s crust, is the fifteenth abundant element on earth (Mirzaee et al. 2020). Natural Sr has four stable isotopes: 84Sr, 86Sr, 87Sr, and 88Sr, and their natural abundances are 0.56%, 9.86%, 7.00%, and 82.58%, respectively (Lide 1995). Due to the dissolution of its natural compounds, Sr can be found in air, soil, and water (Zhang et al. 2018).

There are around 320 mg Sr in our body (Nielsen 2004). Up to 99% of absorbed Sr is stored in the bone, and only 0.7% is dissolved in extracellular fluid (Cabrera et al. 1999). However, it is not clear whether Sr is an essential trace element in our body (Liu et al. 2019). Sr can promote bone growth and prevent and treat osteoporosis (Alexandersen et al. 2011). Several studies showed that Sr was beneficial to secrete cartilage matrix, to stimulate human osteoblast proliferation, to enhance bone mineralization, and to inhibit osteoclast differentiation and resorption (Michael et al. 2015; Cabrera et al. 1999). Strontium ranelate, an organic salt of Sr, has been widely applied in osteoporosis treatment (Rossi et al. 2014). Sr could decrease enamel solubility to prevent the decrease of the hardness of the enamel surface (Wang et al. 2019), so Sr salts are added into toothpaste to maintain dental health. On the contrary, the intake of a high Sr level may pose a potential threat to human health (Langley et al. 2009). For example, an animal experiment on chickens found that a high intake of Sr could affect the synthesis of 1,25-dihydroxycholecalciferol in the kidney; thus, the absorption of calcium was reduced, which ultimately led to rickets (Omdahl and Deluca 1971). As to the group with renal dysfunction, the high intake of Sr has significant health risks (Oste et al. 2005).

The Sr intake in the human body mainly comes from drinking water and food (Greve et al. 2007). As for the adults, in most parts of the world, the total daily intake of Sr is about 4 mg (WHO 2010). Among them, 0.7–2.0 mg is from drinking water, and 1.2–2.3 mg is from food (leafy vegetables, grains, and dairy products) (WHO 2010; Yekta and Sadeghi 2018). However, during the process of food washing and cooking, Sr in drinking water could be absorbed by the negatively charged polymers (protein and polysaccharide) in food. As a result, the Sr in drinking water is crucial to the Sr balance in our body. At present, the WHO has not established a standard value of Sr in drinking water (WHO 2017). In 2012, the USA released *Edition of the Drinking Water Standards and Health Advisories*, which reported that the reference dose (RfD) of Sr was 0.6 mg/kg/day and the recommended value for lifetime health was 4.0 mg/L (USEPA 2012). In October 2014, the United States Environmental Protection Agency announced a regulatory decision on Sr in drinking water and set the health reference level (HRL) of Sr at 1.5 mg/L (USEPA 2014). Currently, Sr is not a restrictive indicator in China (Jin et al. 2006). Based on the *National Food Safety Standard Drinking Natural Mineral Water* (GB 8537–2018), which was released in 2018, the lower limit of Sr is 0.2 mg/L, and there is no upper limit. Therefore, Sr is excluded from water quality monitoring. It is urgent to obtain the Sr concentration in public drinking water in major cities in China.

Besides, the studies about the relationship between Sr content in drinking water and body health in China are limited. The correlation between Sr concentration in drinking water and some diseases has been proven (Curzon et al. 1978; Dawson et al. 1978). Curzon et al. (1978) found that Sr in drinking water could prevent caries when they did an epidemiological investigation in Wisconsin, USA. They proposed that the incidence of dental caries in community children was the lowest when Sr concentration was 5–6 mg/L in drinking water. Dawson et al. (1978) found that Sr content in drinking water was significantly negatively correlated with the incidence and mortality of cardiovascular disease. Several studies about environmental factors of longevity reported that there was a positive correlation between Sr in drinking water and longevity (Liu et al. 2018; Lv et al. 2011). Therefore, it is necessary to study the relation between Sr in drinking water and diseases in China; for instance, we have no conception of the Sr concentration versus BMD and rickets.

Now that the Sr concentration in drinking water has a relationship with health issues, it is significant to conduct the health risk assessment of Sr. Khandare et al. (2020) carried out a health risk assessment of Sr in drinking water in 58 villages in India and proposed that the hazard index (HI) values of adults in 45 villages and children in 56 villages were above 1, which meant there existed obvious non-carcinogenic risk. During the process of evaluation, they did not consider the amount of drinking water and the difference in body weight, which made the results of the assessment uncertain. As to Chinese cities, Only Zhang et al. (2018) did a health risk assessment in Xi’an city. Due to the fact that China has a vast territory, there are great differences in water quality in different regions. As a result, the relationship between Sr concentration in public drinking water and health risk is still unclear.

At this moment, drinking water problems in China are quite complex. On the one hand, the potential threats posed by high concentrations of trace elements in drinking water are increasing; on the other hand, water purifiers based on reverse osmosis technology are widely used in Chinese families, which results in the lack of trace elements that maintain the body health in drinking water. As to Sr, the relationship between Sr and body health and its health risk evaluation are the problems to be solved immediately in public drinking water management. In this research, public drinking water was sampled and analyzed in the major cities in China to understand the content and spatial distribution of Sr and to evaluate the contribution of drinking water to the total Sr intake and potential health risks in the human body. The research results will help us to understand the quality of public drinking water in Chinese cities and to provide a reference for making standards of drinking water and risk management.

## Materials and methods

### Sampling and analytical procedures

From December 2019 to January 2020, we planned to collect water samples from 337 prefecture-level cities in China. Due to the low temperature (< − 10 °C) in many parts of China during that time, it was not available to get some samples from remote cities in Tibet and Xinjiang. Finally, we collected 314 water samples from 314 cities (the names of cities are shown in Table S1). As China is vast in the territory, the geological conditions, climate conditions and water quality conditions have significant differences in different regions. Therefore, this research was conducted based on geographical division. According to the characteristics of geological and climate conditions, China could be divided into four regions; namely, northern China (NC), southern China (SC), northwest China (NWC), and Qinghai-Tibet Plateau area (QT), among which, Qinling mountain-Huai river is the boundary between SC and NC, and Daxinganling Mountains-Yinshan Mountains-Helan Mountains is the mark between NC and NWC. As shown in Fig. S1, we collected 108 samples from NC, 163 samples from SC, 31 samples from NWC, and 12 samples from QT. Fewer samples were obtained in the NWC, QT, and the northeast part of NC mainly due to the harsh natural conditions, low population density, and fewer cities in these areas.

All the samples were taken from residential dwellings. Before sampling, we had confirmed that the water source was the local public water supply company. In addition, all the water samples were not filtered by household water purifiers or reverse osmosis systems. The sampling procedure was as follows: First, we turned on the water tap and let the water flow out for at least 5 min to drain retained water in the pipe network. Then, four 500-ml PET bottles, which were acid pre-cleaned (5% HCl), were used to collect water after washing three times with sampling water. Finally, these bottles were sealed by sealant when there were no air bubbles in the bottle. All the water samples were collected between 10 am and 4 pm and were sent to the laboratory by express courier within 36 h. Ice bags were placed with the water samples to maintain the ambient temperature below 8 °C during transportation. All samples were processed immediately after delivery. Conductivity and pH of water in four PET bottles were tested at the same time. Some water samples, whose transportation time was more than 36 h, or the difference of conductivity among four PET bottles was more than 5%, or air bubbles existed in the bottles, were judged as unqualified samples, so we had to resample them. For the qualified samples, they were filtered by the 0.45-μm filter membrane (Sartorius Minisart, Hannover, Germany). After filtration, the water was collected by a 50-ml acid pre-cleaned high density polythene (HDPE) bottle, and strong guaranteed nitric acid was added into the water sample until the pH of the water was below 2 for cation measurement. All the water samples were preserved in the cold room (4 °C) for the next measurement.

Based on the national standard (HJ 700–2014) proposed by the ministry of environmental protection of China (MEPC 2014), Sr concentration in water samples was determined by the inductively coupled plasma emission mass spectrometer (ICP-MS, Agilent 7700, Agilent). In addition, in order to obtain the calcium-strontium ratio and elucidate the relationship between Sr and total hardness, the concentrations of calcium and magnesium were measured at the same time. In order to control the quality of measurement, besides the initial calibration, a new calibration curve was established after measuring every 10 samples. A standard solution (8500–6940, Agilent) was applied to check the accuracy of ICP-MS measurement. The recovery rates of standard samples were in the range of 90–110%. In addition, the relative standard deviations of the samples were all below 10%.

**Fig. 1:**
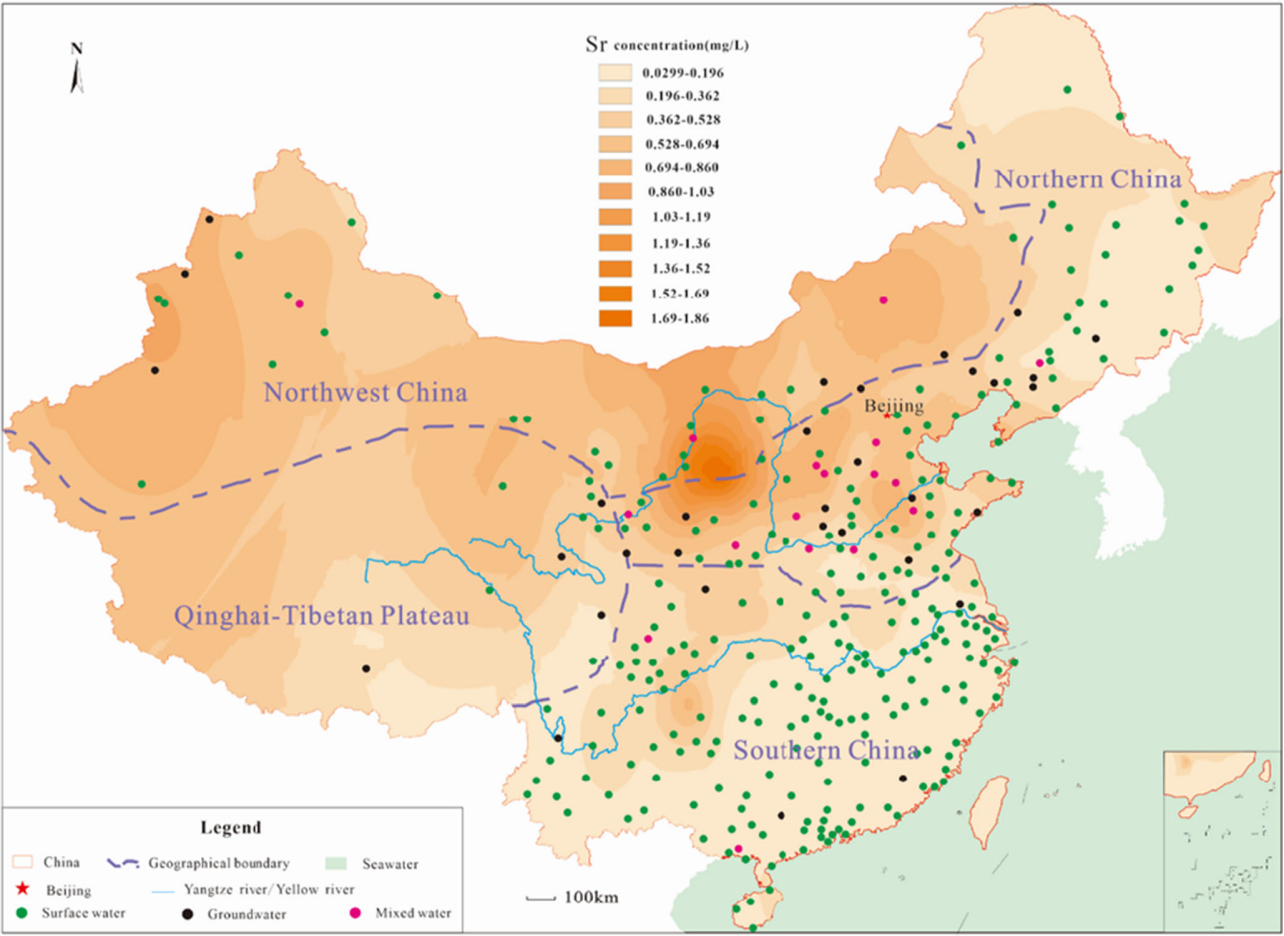
The distribution of strontium concentrations in public drinking water in China

The Sr concentrations in the different cities were statistically analyzed by SPSS (IBM, 23.0 edition). As the data did not follow the normal distribution or logarithmic distribution, the Kruskal-Wallis non-parametric test was applied to check the differences in Sr concentrations in different cities. According to the two-tailed test, the effects were considered statistically significant with *p* < 0.05. Anderson-Darling test was conducted for the goodness of fit test. On this basis, according to the distribution of Sr concentration in public drinking water in different cities and the distribution of the amounts of drinking water in different age groups, Monte-Carlo (MC) simulations were conducted to calculate the average amount of Sr from drinking water in different age groups and different cities. In addition, the spatial distribution of Sr was drawn by ArcGIS (10.5 edition) based on the inverse distance weighting (IDW) model (see Fig. 1).

### Epidemiological investigation of rickets and bone mineral density

In order to obtain the effects of Sr in drinking water on body health, the correlation analyses between Sr concentration and rickets and bone mineral density were conducted. Bone mineral density is a standard index for clinical diagnosis of osteoporosis (McDonough et al. 2008). Only a few studies have reported nationwide rickets and osteoporosis prevalence in China (Strand et al. 2007; Cui et al. 2020). So we took advantage of the China National Knowledge Infrastructure (CNKI) database and Wanfang database to obtain 136 research papers about the epidemiological survey on the incidence of rickets among children in China and 125 research papers about the epidemiological survey on the bone mineral density of middle-aged and elderly adults in China. Due to the differences existing in the chosen population characteristics and assessment methods, it is difficult to directly compare the data from the research papers. As a result, we filtered the data based on epidemiological methods. Finally, the incidence of rickets among children was obtained from the studies about 0*–*3 years age group in 48 cities (see Table S2). These studies all took the examinations of bone X-ray or bone alkaline phosphatase reagent (BALP). Although the incidence of rickets diagnosed by BALP is a little bit higher than that diagnosed by X-ray, the difference does not have a significant impact on data analysis (Taylor et al. 2010). The bone density was from papers about 60*–*70 years age group in 31 cities (see Table S3). SPSS (IBM, 23.0 edition) was applied to calculate Spearman’s correlation coefficients among Sr concentration, rickets, and bone mineral density.

### Exposure and health risk appraisal

Health risk assessment (HRA) is a conventional model that evaluates the possible harmful effects by contacting specific chemical and microbiological reagents within a particular period. HRA has been widely applied in the quantification of potential risk (Dong et al. 2020; Long and Luo 2020; Xiao et al. 2019; Zhang et al. 2020). In this research, based on physiological and social change, the total population was divided into four age groups: infants (age 1–4 years), children (age 5–10 years), teens (age 11–20 years), and adults (age 21–72 years) (Fallahzadeh et al. 2018; Rahman et al. 2020). The purpose of this division was to understand the effects of Sr exposure on different age groups and estimate its potential non-carcinogenic health risks. Related potential health risks of these four groups were evaluated respectively.

The primary exposure pathways of Sr to humans are dermal contact (e.g., taking a bath) and oral intake (e.g., drinking water). In this research, the hazard index was applied to evaluate health risks from the aspects mentioned above.

The health risk caused by oral intake was calculated as follow (Long and Luo 2020; Xiao et al. 2019):

1 $$ {\mathrm{HQ}}_{\mathrm{i}}=\frac{C_w\times \mathrm{DR}\times {\mathrm{EF}}_{\mathrm{i}}\times {\mathrm{ED}}_{\mathrm{i}}}{\mathrm{BW}\times \mathrm{AT}\times {\mathrm{RfD}}_{\mathrm{i}}} $$

where *HQ*_i_ is the ingestion hazard quotient; *C*_*w*_ is the measured concentration of Sr (mg/L); *DR* is the water consumption rate (L/d); *EF*_i_ is the ingestion exposure frequency (d/y); *ED*_i_ is the ingestion exposure duration (y); *BW* is the body weight (kg); *AT* is the average time for non-carcinogens (d); and *RfD*_i_ is the reference dose of Sr via oral exposure pathway (mg/kg/day).

The following equation calculated the health risk caused by dermal contact (Long and Luo 2020, Xiao et al. 2019):

2 $$ {\mathrm{HQ}}_{\mathrm{d}}={\mathrm{K}}_{\mathrm{p}}\times {C}_w\times \frac{\mathrm{ET}\times {\mathrm{ED}}_{\mathrm{d}}\times {\mathrm{EF}}_{\mathrm{d}}\times \mathrm{SA}\times {10}^{-3}}{\mathrm{BW}\times \mathrm{AT}}\times \frac{1}{{\mathrm{RfD}}_{\mathrm{d}}\times \mathrm{GIABS}} $$

where *HQ*_d_ is the dermal hazard quotient; *K*_p_ is the dermal permeability coefficient of pollutant (cm/h); *ET* is the exposure time (h/d); *ED*_d_ is the dermal contact exposure duration (y); *EF*_d_ is the dermal contact exposure frequency (d/y); *GIABS* is the fraction of chemical absorbed in the gastrointestinal tract (its value is 1); *SA* is the exposed skin area (cm^2^); 10^−3^ is the volume conversion factor (L/cm^3^); and *RfD*_d_ is the reference dose of the Sr via dermal pathway (mg/kg/day).

The total hazard index (HI) via multiple exposure pathways could be calculated as follow:

3 $$ \mathrm{HI}={\mathrm{HQ}}_{\mathrm{i}}+{\mathrm{HQ}}_{\mathrm{d}} $$

When HI < 1, the adverse health effects could be ignored; when HI > 1, it suggests a potential non-carcinogenic risk.

### Monte-Carlo simulations and sensitivity analysis

The non-carcinogenic risk assessment was carried out to evaluate the health risks of Sr in public drinking water in Chinese cities. The fitting input variables presented in Table 1 were used in the Monte Carlo simulation to estimate the exposure risk. What’s more, the hazard quotient and hazard index of Sr through the oral intake and skin absorption were obtained. Taking advantage of the Monte-Carlo method (Malakootian et al. 2020), 1000 repeated simulations were carried out with hypercube to conduct the probabilistic analysis on the uncertainty during the risk assessment. Assumed variable distribution provided by Wu et al. (2011), Zhang et al. (2017), and USEPA (2004) evaluated the health risks of Sr among different age groups (see Table 1). Appropriate parameters of Chinese population characteristics (such as drinking water ingestion rate (DR), skin surface area (SA), and body weight (BW)) would decrease the uncertainty of evaluation. Subsequently, crystal ball 11.1 (Oracle Inc., American) was employed to do simulation analysis. During the simulation process, we could recognize the importance of the input parameters by sensitivity analysis. The output parameters of sensitivity analysis were defined as correlation coefficients. When the coefficients were larger, the greater influence caused by input parameters was exerted on the calculated risks (Yin et al. 2019).

**Table 1:** Summary of the representative values or distributions for the input parameters

Parameter	Unit	Parameter unit representative value or distribution ^a^				References
		Infants	Children	Teens	Adults	
DR (drinking water ingestion rate)	L/d	Normal (0.75, 0.08)	Normal (1.25, 0.57)	Normal (1.58, 0.69)	Normal (1.95, 0.64)	Zhang et al. (2017)
EF (exposure frequency)	d/y	350	350	350	350	Wu et al. (2011)
ED (exposure duration)	y	Uniform (0, 10)^b^	Uniform (0, 10)	Uniform (0, 10)	Uniform (0, 50)	Wu et al. (2011)
BW (body weight)	kg	Lognormal (6.00, 0.60)	Lognormal (16.68, 1.48)	Lognormal (46.25, 1.18)	Lognormal (57.03, 1.10)	Wu et al. (2011); Zhang et al. (2017)
AT (average lifespan)	d	2190	2190	2190	9125	MEPRC (2014)
SA (skin surface area)	cm^2^	Lognormal (7422, 1.25)^b^	Lognormal (7422, 1.25)	Lognormal (14,321, 1.18)	Lognormal (18,182, 1.10)	Zhang et al. (2017)
ET (exposure time)	h/d	Lognormal (0.33, 1.89)^b^	Lognormal (0.33, 1.89)	Lognormal (0.27, 1.24)	Lognormal (0.25, 1.26)	Wu et al. (2011)
K_P_ (permeability coefficient)	cm/h	1.0 × 10^−3^	1.0 × 10^−3^	1.0 × 10^−3^	1.0 × 10^−3^	USEPA (2004)
RfD_d_ (reference dose)	mg/kg/day	0.12	0.12	0.12	0.12	Xiao et al. (2019)
RfD_i_ (reference dose)	mg/kg/day	0.6	0.6	0.6	0.6	Xiao et al. (2019)
GIABS (absorption fraction in GIT)	Unitless	1	1	1	1	USEPA (2016)

^a^Normal (arithmetic mean, standard deviation); lognormal (geometric mean, geometric standard deviation); uniform (minimum value, maximum value)

^b^There is no data on infant distribution, so the data on child distribution substitute

## Results

### Concentrations of strontium in all of the samples

Sr was detected in water samples from 314 cities (see Table S1). Table 2 presented the Sr levels in drinking water in Chinese cities. In general, the range of Sr concentration was 0.005–3.11 mg/L with a mean value of 0.360 mg/L. Compared with Sr concentration in the southwest part of Cairo (mean value 0.867 mg/L) and the USA (mean value 1.10 mg/L), it was relatively low in Chinese cities. As shown in Fig. S2, there were 295 cities whose Sr concentration mainly fell in the range of 0.005–1 mg/L. Thus the number of these cities accounted for 93.95% of the total number of cities (314 cities). Among them, there were 76 cities whose Sr concentration was below 0.1 mg/L, which occupied 24.2%; the number of cities with Sr concentration of 0.1–0.2 mg/L and 0.2–0.3 mg/L was 61 and 60, respectively.

**Table 2:** Strontium concentrations in public drinking water in Chinese cities (mg/L)

Sampling zone^a^	N^b^	Mean	SD^c^	Max^d^	Min^e^	Median
Overall	314	0.360	0.385	3.11	0.005	0.008
NC	108	0.537	0.483	3.11	0.051	0.392
SC	163	0.179	0.182	1.59	0.005	0.127
NWC	31	0.667	0.333	1.43	0.041	0.639
QT	12	0.439	0.320	1.08	0.094	0.315

^a^*NC*, Northern China; *SC*, Southern China; *NWC*, Northwest China; *QT*, Qinghai-Tibet Plateau

^b^Number of samples

^c^Standard deviation

^d^Maximum

^e^Minimum

As presented in Table 2, Sr concentrations were the highest in NWC with a range of 0.041 to 1.43 mg/L, mean of 0.667 mg/L; the lowest Sr concentration was detected in SC with a range of 0.005–1.59 mg/L, mean of 0.179 mg/L. The Sr concentration in NC and QT was 0.051–3.11 mg/L with an average of 0.537 mg/L and 0.094–1.08 mg/L with an average of 0.439 mg/L, respectively. It could be found in Fig. S3 that Sr concentration in public drinking water in different Chinese cities followed the sequence NWC > NC > QT > SC. According to the Kruskal-Wallis non-parametric test, the concomitant probability value was 0, which meant the relationship of Sr concentrations between different cities was statistically significant.

In Fig. 1, it was clear to see that the Sr concentration in public drinking water in Chinese cities presented geographically aggregated distribution. Sr level was relatively low in the southern region of the Yangtze River. Sr concentration was below 0.1 mg/L in most cities except the middle part of Guizhou province (Sr concentration was above 0.5 mg/L). Sr concentration in the cities along the Yangtze River was mostly between 0.2 and 0.3 mg/L. Cities in which Sr concentration was above 0.5 mg/L were found in the Yellow River Basin and Xinjiang. As shown in Fig. 1, Sr concentration was higher in the northwest than in the southeast.

### The intake of strontium through drinking water

**Fig. 2:**
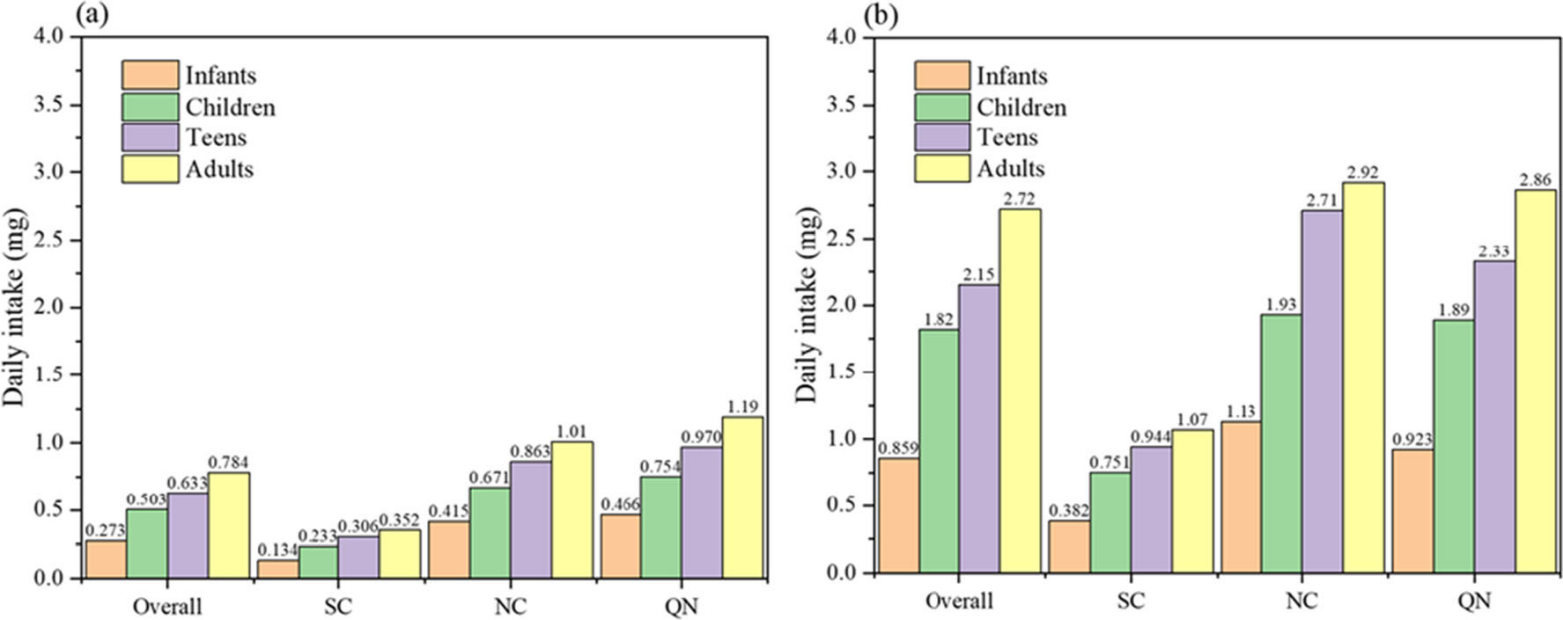
The Sr intake through drinking water in different age groups in different regions: (a) mean values; and (b) 95th percentile values (NC, Northern China; SC, Southern China; QN, Northwest China and Qinghai-Tibet Plateau)

The average Sr concentrations intaking through drinking water in different age groups in different regions were shown in Fig. 2a. In general, the average Sr intakes of infants, children, teens, and adults through public drinking water were 0.273, 0.503, 0.633, and 0.784 mg/day, respectively, which was below the daily intake of Sr from drinking water in the USA (2 mg/L) (WHO 2010). In terms of geographical distribution, the intake of Sr via public drinking water was the lowest in SC, which was half of the national average intake of Sr. However, in the QN (the combination of QT and NWC), the Sr intake by drinking water was the highest, and the Sr intake in adults could reach 1.19 mg/day. At present, the total daily intake of Sr in China is not clear. Nevertheless, we know the total daily intakes of Sr in Japan, Finland, and the USA are 2.3 mg/day, 1.91 mg/day, and 3.3 mg/day, respectively (Shiraishi et al. 1994; Varo et al. 1982; WHO 2010). Assuming that the total daily intake of Sr in China is between Finland and the USA, namely, the daily intake of Sr in China is estimated in the range of 1.91–3.3 mg/day. Therefore, based on the intakes from various countries, the Sr intake from public drinking water accounts for 24–41% of the total daily intake of Sr for an adult in China. As you see, public drinking water is one of the most important ways to get Sr. In Fig. 2b, the 95th percentile value of the national average daily intake of Sr through drinking water was 2.72 mg for the adults, especially in NC and QN which values were 2.92 mg and 2.86 mg, respectively. As a result, for the cities which have a relatively high Sr concentration in NC and QN, drinking water is the main source to intake Sr. Although there are some studies reported the Sr content in human urine, the Sr content in feces and sweat and the amount of Sr retained in the bones are unclear (Yang et al. 2019). So it is still difficult to determine the estimated average (daily) requirement (EAR) and recommended daily intake (RDA) of Sr.

### The correlation between strontium in drinking water and human disease

Although Sr is a trace element having a high concentration in drinking water and the mineral water containing high Sr concentration is quite popular among customers (Misund et al. 1999), the studies about the relationship between Sr content and body health are limited. A previous study proposed that Sr content in drinking water was significantly negatively correlated with the incidence and mortality of cardiovascular diseases. However, as shown in Fig. S4, the cities having a relatively high Sr concentration in drinking water also have a relatively high total hardness. It is widely accepted that the hardness of drinking water could prevent cardiovascular diseases to some extent (Monarca et al. 2003). As a result, the relationship between Sr concentration and cardiovascular diseases is still being concerned.

**Fig. 3:**
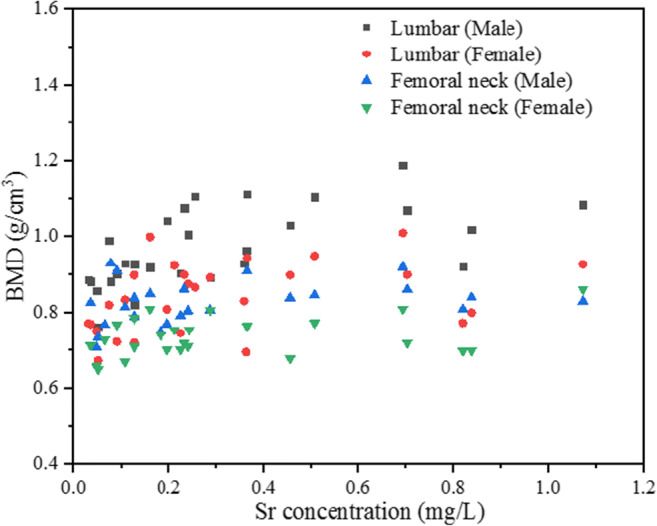
Scatter plot of bone mineral density (BMD) of 60–70 years old residents vs. Sr concentration in public drinking water in 31 cities in China

Moreover, Sr has been utilized in the prevention of osteoporosis (Alexandersen et al. 2011). Figure 3 presented a significant correlation between the bone mineral density (BMD) of the 60–70 years older people and Sr concentration in drinking water. For example, the elderly people from the cities with a lower Sr concentration had a lower BMD in the lumbar spine and femoral neck bone. As presented in Table 3, the correlation between Sr concentration in drinking water and the BMD of the lumbar spine was stronger than that between Sr concentration and the BMD of the femoral neck bone. Besides, the correlation coefficient between Sr concentration and male elderly people was 0.692 (*p* < 0.01); meanwhile, the correlation coefficient of female elderly people was 0.483 (*p* < 0.01), which presented a significantly positive correlation.

**Table 3:** The correlation between bone mineral density (BMD) and Sr as well as that between BMD and Ca in public drinking water

Element	Lumbar BMD (male)	Lumbar BMD (female)	Femoral neck BMD (male)	Femoral neck BMD (female)
Sr	0.692^**^	0.483^**^	0.351	0.293
Ca	0.577^**^	0.478^**^	0.112	0.343

^**^significantly correlated at 0.01 level of probability

**Fig. 4:**
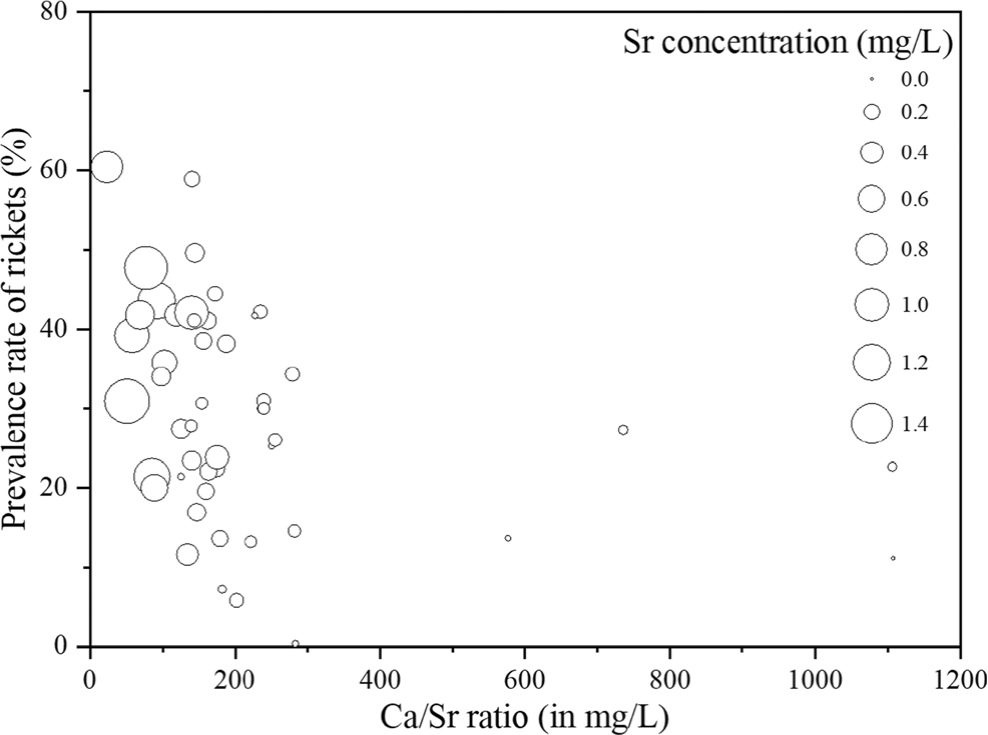
Scatter plot of correlation between prevalence rate of rickets of 1–3 years old children, Sr concentration and Ca/Sr ratio in public drinking water in 48 cities in China

As shown in Fig. 4, it is clear to see that the high incidence of rickets in children was found in the cities with high Sr concentration and low Ca/Sr ratio in drinking water. Besides, among the age group of 1–3 years, the correlation coefficient of Sr concentration and the incidence of rickets was 0.411 (*p* < 0.05), while there was a negative correlation between Ca/Sr ratio and the incidence of rickets with a correlation coefficient of − 0.410 (*p* < 0.05).

### Health risk assessment

**Fig. 5:**
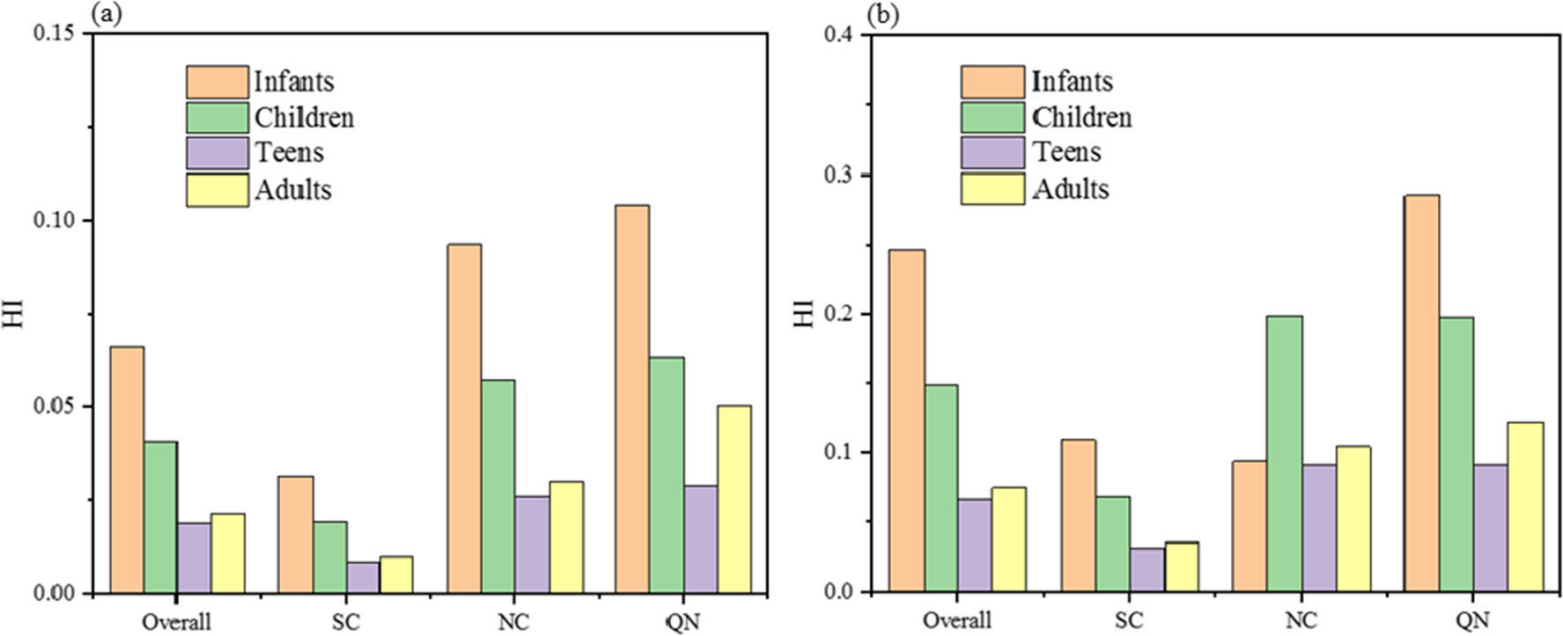
Hazard index distribution in different age groups in different regions: (**a**) mean values; and (**b**) 95th percentile values (NC, Northern China; SC, Southern China; QN, Northwest China and Qinghai-Tibet Plateau)

The hazard quotient and hazard index of Sr through the oral intake and skin absorption in different age groups in different regions are shown in Table S4. The mean value of *HQ*_d_ and 95th percentile values were in the order of 10^−4^–10^−3^; the average value of *HQ*_i_ and 95th percentile values were mostly in the order of 10^−1^–10^−2^. *HQ*_i_ was two orders of magnitude higher than *HQ*_d_. Therefore, oral intake was the main exposure route of Sr in public drinking water. In general, the mean value of HI and 95th percentile values of exposed Sr in public drinking water in Chinese cities were all less than 1, so the non-carcinogenic risk of exposed Sr was not obvious. Among people of different ages, the infants’ HI was the highest (average value 0.066; 95th percentile value 0.247), followed by the children’s HI (average value 0.041; 95th percentile value 0.149) and adults’ HI (average value 0.021; 95th percentile value 0.075). The teens’ HI was the lowest (average value 0.019; 95th percentile value 0.066). The infants’ HI was nearly two times higher than that of the children, and the HI of the children was almost two times than that of the teens; the HI of the teens was close to that of the adults. In Fig. 5a and Fig. 5b, HI of four age groups in SC was the lowest; the mean values of HI and 95th percentile values were one time lower than the national average level. HI of four age groups in NC and QN were all above the national average level. The mean values of HI and the 95th percentile values of four age groups in QN were all higher than those in NC. In Table S4, however, the maximum value of infants’ HI in NC was 0.795, which was close to the theoretical threshold of risk. Therefore, it was necessary to conduct detection on Sr content in the NC where there was a high Sr concentration in public drinking water.

The results of sensitivity analysis were presented in Fig. S5. Except for the cities in QN, Sr concentration (*Cw*) and exposure duration (*ED*) had the most significant influence on different age groups, and their correlation coefficients were 0.466–0.667 and 0.276–0.389. The drinking water ingestion rate (*DR*) has a significant impact on the children, teens, and adults (their correlation coefficients fell in the range of 0.077–0.177), but its influence on infant could be ignored. In QN, Sr concentration (*Cw*) and drinking water ingestion rate (*DR*) were the most influential factors on the output risk of adults, and the exposure duration (*ED*) was a minor factor. The results of the sensitivity analysis indicated that the better definition of the probability distribution of *C*_*w*_, *ED*, and *DR* could obtain a more accurate risk evaluation.

## Discussion

### Factors affecting the spatial distribution of strontium concentration

The characteristics of the geographic distribution of Sr content were related to the geological environment. The main source of Sr in surface water and groundwater is from water-rock interaction. Sr usually exists in the rocks, such as carbonate rocks and clastic rocks, in the form of strontium sulfate and strontium carbonate. Sr content in different rocks follows the order carbonate rocks > clastic rocks > metamorphic rocks > extruded rocks > cenozoic loose rocks > intermediate-acid intrusive rocks. Thus, there was a good correlation between the Sr and Ca and Mg in groundwater and surface water. For instance, in Fig. S4 and Table S5, Sr concentration had a positive correlation with the total hardness (*r* = 0.868, *p* < 0.01). Overall, the higher the total hardness, the higher the Sr concentration. In the Yangtze River Basin, Sr concentration in surface water is related to the weathering of celestite and strontianite in the dolomite. Although a large area of limestone is distributed in the south of the Yangtze River, the Sr level is relatively low in this rock due to the effect of the sedimentary environment. As a result, at the same total hardness, Sr concentration in the south is lower than that in the north and northwest. Sr concentration is relatively high in north and northwest cities because there were a large number of carbonate rocks and pyroclastic rocks in which Sr content is high. Besides, the north is arid and rainless. Both aspects lead to the accumulation of Sr in the groundwater and surface water. For example, compared with the distribution map of karst terrain, the karst area in NC has relatively high Sr concentrations at the same time (Liang et al. 2018). In Fig. 1, among the sources of public drinking water, the water sources consisted of groundwater and surface water are more common in the north; however, drinking water mainly comes from surface water in the south. Therefore, the water-rock interaction taken place in groundwater is more intensive in the north resulting in the Sr accumulation.

Because there were few sampling points in QT, besides, QT and NWC were sparsely populated areas and had few cities, the total number of people only accounts for 5% of the total population of China. Thus, we combined QT and NWC for the analysis when we discussed the contribution of Sr in drinking water to the total Sr intake in our body and its health risks (QN was used in place of the combination of QT and NWC). The results of the goodness of fit test showed that Sr concentration in drinking water in NC and SC was in accord with Gamma distribution, Sr concentration in QT and NWC fit the Beta distribution. Sr concentration in the whole country conformed to the lognormal distribution. Table S6 listed the obtained distributed parameters.

### The effects of Sr concentration in drinking water on body health

At present, it has been proven that Sr could contribute to secrete cartilage matrix, to stimulate human osteoblast proliferation, and to enhance bone mineralization. As a result, the bone mineral density is increased (Alexandersen et al. 2011; Cianferotti et al. 2013; Michael et al. 2015). It is generally believed that the calcium (Ca) in drinking water is the main factor affecting bone mineral density (Costi et al. 1999). However, also shown in Table 3, the correlation coefficients of Sr were higher than that of Ca (except for the femoral neck BMD (Female)). Ca and Sr have synergism effects on maintaining bone health. Sr could activate the Ca-sensing receptor (CaSR), which plays an important role in the formation of osteoblasts and osteoclasts (Pi and Quarles 2004). As a result, the Sr in drinking water cannot be ignored in the prevention of osteoporosis.

Even though the Sr does good to the bone health, long-term drinking of water having a relatively high Sr concentration will affect the bone mineralization (Omdahl and Deluca 1971). The high Sr intake will influence the synthesis of 1,25-dihydroxyvitamin D3 and the Ca absorption in the intestine(Omdahl 1977). Besides, Sr can replace the Ca within the hydroxyapatite of bone by ion exchange, which results in the decrease of bone calcium. Thus the disease caused by the bone mineralization defects is induced, such as rickets which is a common bone disease in infants and children (Cabrera et al. 1999). As a result, when the Sr concentration in drinking water is larger, the BMD of the elderly is larger and the rickets incidence of the children is higher. This is consistent with the result of epidemiological investigation of rickets and bone mineral density in our study.

**Fig. 6:**
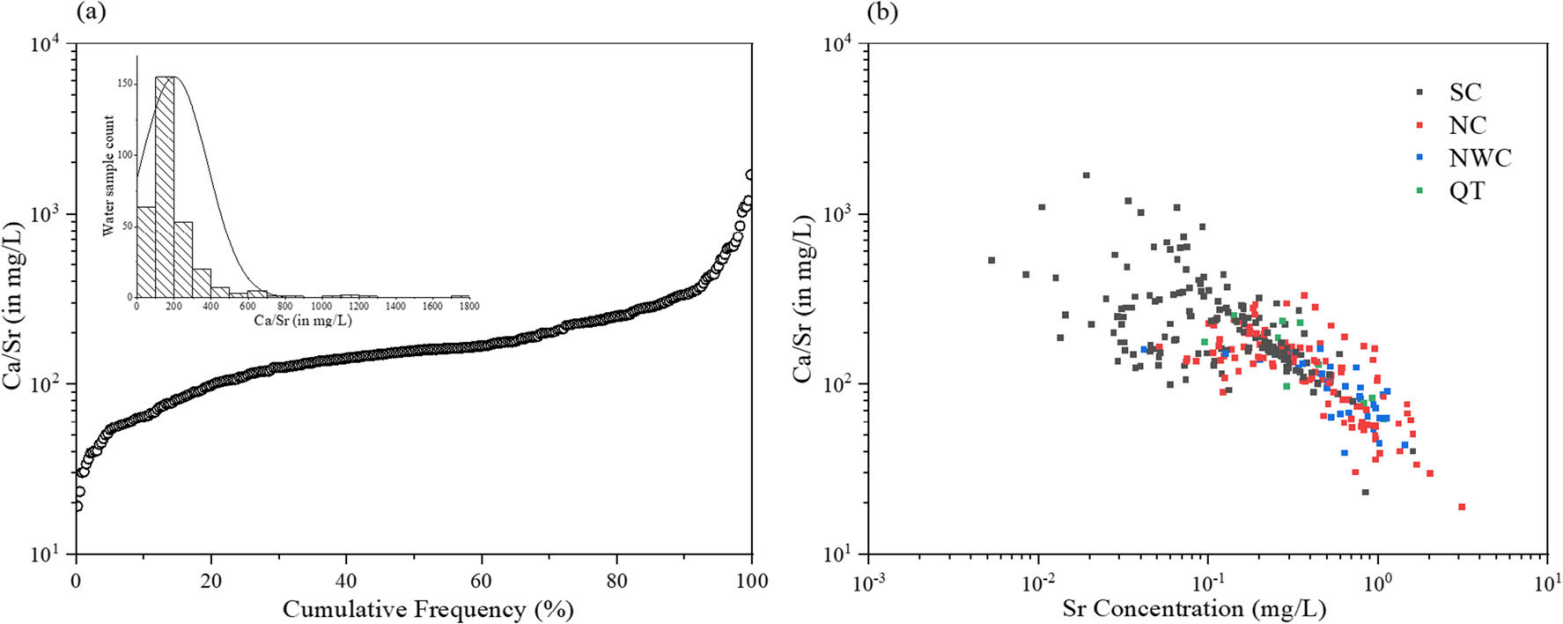
**a** Probability distribution of calcium-strontium ratio in public drinking water in China. **b** The relationship between strontium concentration and calcium-strontium ratio in drinking water samples from different regions (NC, Northern China; SC, Southern China; NWC, Northwest China; QT, Qinghai-Tibet Plateau)

Several studies also reported that there was a competition absorption between Ca and Sr and Ca/Sr ratio strongly influenced bone deformity; when Ca/Sr ratio decreases, the risk will increase, vice versa (Grynpas et al. 1996; Storey 1961; Zeneli and Daci 2014). Therefore, the range of Ca/Sr ratio in drinking water is critical for bone health, but the fitting range is not clear at present. As shown in Fig. 6a, the Ca/Sr ratios in the Chinese cities fell in the range of 19.2–1700 (its mean value was 199). The number of cities whose ratios were below 100 was 64, which accounted for 20.4%. There were 155 cities where the ratios were between 100 and 200; it accounted for 49.4%. In Fig. 6b, the water samples with the larger Ca/Sr ratios were distributed in the south; the Sr concentration was relatively low at the same time. However, the water samples with small Ca/Sr ratios were found in both north and northwest regions, and the Sr levels were also high in those regions.

### Limitations

Although the uncertainty of health risk evaluation of Sr was quantified by Monte Carlo simulation, there were still some limitations and uncertainties in this research. For example, a reference dose (RfD) of Sr (0.6 mg/kg/day) was obtained by the no observed adverse effect level (NOAEL = 190 mg Sr/kg/day) and uncertainty factors (UF = 300). However, the NOAEL was obtained by animal experiments (Storey 1961). The young rats and adult rats were used as the experiment objects. Strontium carbonate, as a Sr source, was added to the food which contained 1.6% calcium source as well. Then NOAEL was measured by the dysplasia of the epiphysis of the rats. However, Ca and Sr have synergistic and antagonistic effects; low Ca and high Sr are more likely to induce dysplasia of the epiphysis. At the same time, bicarbonate would also reduce bone resorption (Rylander 2008). As a result, it was more accurate to get NOAEL using food that contained strontium sulfate but did not add calcium. In addition, except for drinking water, Sr from food also made a significant contribution, especially in seafood which contained up to 25 mg/kg of Sr (Nabrzyski and Gajewska 2002). Besides, cooking with drinking water containing a high Sr concentration would increase the intake of Sr as well (Melnyk et al. 2019). As a result, Sr exposure from food was not considered in this research, which might lead to the underestimation of the non-carcinogenic risk of Sr exposure. Thus, we should establish the management and control of Sr in drinking water in NC and QN cities where the Sr concentration was relatively high and the Ca/Sr ratio was relatively low.

## Conclusions

In this study, we attempt to provide information on the Sr concentration in public drinking water in Chinses cities, the intake of Sr from drinking water, and the Sr health risks. The Sr concentrations of public drinking water fall in the range of 0.005–3.11 mg/L with a mean of 0.360 mg/L. There are obvious differences in the Sr concentration in different cities. Overall, Sr concentration is higher in the north than in the south, among which the NWC has the highest Sr concentration (0.041–1.44 mg/L, its mean value 0.667 mg/L). The lowest Sr concentration was found in the SC (0.005–1.59 mg/L, its mean value 0.179 mg/L). The average Sr intakes of infants, children, teens, and adults through public drinking water were 0.273, 0.503, 0.633, and 0.784 mg/day, respectively. In terms of geographical distribution, the amount of Sr intake from public drinking water is the smallest in SC but the largest in NC. The daily Sr intake through public drinking water is 24–42% of total daily Sr intake, based on the total daily intake of other countries. The correlation coefficients between Sr concentration and the elderly’s lumbar bone mineral density were 0.692 (*p* < 0.01) for males and 0.483 (*p* < 0.01) for females, which displayed a significantly positive correlation. As a result, more attention should be paid to the Sr effects on osteoporosis prevention. In addition, the cities with a low Sr concentration in public drinking water, especially in SC, should consider changing the public drinking water treatment process. For instance, they can apply new technology to remineralize drinking water to increase the Sr concentration.

The analysis of health risk shows that, among different age groups, the infants’ HI is the highest (average value 0.0663; 95th percentile value 0.247). The non-carcinogenic risk of Sr among different age groups in different regions through drinking water was within acceptable levels (HI ≤ 1). Therefore, Sr in the drinking water has no significant risks to the inhabitants. However, the contribution of food to the total Sr intake and the antagonistic action between Sr and Ca are not considered which may lead to the underestimation of non-carcinogenic risk of Sr. In addition, the incidence of rickets in children presents a significantly positive correlation with Sr concentration (*r* = 0.411, *p* < 0.05) and significantly negative correlation with Ca/Sr ratio (*r* = − 0.410, *p* < 0.05). Therefore, even if the HI is less than 1, the cities with high Sr concentration and low Ca/Sr ratio in public drinking water in NC and NWC regions should be under the management of the public drinking water. In general, the results of this research still provide helpful information for the supervision of Sr in public drinking water in Chinese cities.

## Supplementary information

Supplementary information data to this article can be found in the online version of this article.

ESM 1 (DOCX 743 kb)
