# Supplementary material for: Strontium in public drinking water and associated public health risks in Chinese cities
Source: Environ Sci Pollut Res Int. 2021 Jan 12;28(18):23048–59. doi: 10.1007/s11356-021-12378-y (PMC8113192; doi:10.1007/s11356-021-12378-y)
Supplement: Supplementary file 1 — (DOCX 743 kb) [file 11356_2021_12378_MOESM1_ESM.docx]

**Supplementary Material Cover Sheet**

Strontium in public drinking water and associated public health risks in Chinese cities

Hao Peng ^1^, Feifei Yao ^2^, Shuang Xiong ^3^ , Zhonghua Wu ^2^, Geng Niu ^3^ Taotao Lu ^4^ *

^1^ School of Environmental Studies, China University of Geoscience, Wuhan, 430078, China

^2^ Wuhan Zondy W&R Environmental Technology Co. LTD, Wuhan, 430078, China

^3^ Qingdao Haier Smart Technology R&D Co., Ltd, Qingdao, 266101, China

^4^ Department of Hydrology, University of Bayreuth, Bayreuth, 95440, Germany

Manuscript prepared for *Environmental Science and Pollution Research*

* Corresponding author: Taotao Lu (Taotao.Lu@uni-bayreuth.de)

Number of pages: 24

Number of tables: 6

Number of figures: 5

**Table S1** Sr concentrations of 314 cities in China

| Sample number | City’s name | Region | Sr conc^a^  (mg/L) | Sample number | City’s name | Region | Sr conc^a^  (mg/L) |
| --- | --- | --- | --- | --- | --- | --- | --- |
| 1 | Chongqing | SC | 0.3794 | 158 | Hezhou | SC | 0.0475 |
| 2 | Luzhou | SC | 0.2441 | 159 | Fangchenggang | SC | 0.0052 |
| 3 | Dazhou | SC | 0.5201 | 160 | Laibin | SC | 0.2408 |
| 4 | Guang’an | SC | 0.4548 | 161 | Chongzuo | SC | 0.0596 |
| 5 | Zhengzhou | NC | 0.1491 | 162 | Nanjing | SC | 0.2331 |
| 6 | Luoyang | NC | 0.6333 | 163 | Zhenjiang | SC | 0.2431 |
| 7 | Nanyang | SC | 0.1462 | 164 | Yangzhou | SC | 0.2782 |
| 8 | Xinxiang | NC | 0.3935 | 165 | Nanchang | SC | 0.0774 |
| 9 | Xinyang | SC | 0.1185 | 166 | Ganzhou | SC | 0.0367 |
| 10 | Kaifeng | NC | 0.8047 | 167 | Jiujiang | SC | 0.2923 |
| 11 | Shangqiu | NC | 0.9569 | 168 | Shangrao | SC | 0.0143 |
| 12 | Anyang | NC | 0.2147 | 169 | Yichun | SC | 0.2523 |
| 13 | Pingdingshan | NC | 0.2688 | 170 | Fuzhou | SC | 0.0451 |
| 14 | Zhumadian | NC | 0.1836 | 171 | Ji’an | SC | 0.0512 |
| 15 | Jiaozuo | NC | 0.1615 | 172 | Jingdezhen | SC | 0.0515 |
| 16 | Zhoukou | NC | 0.1411 | 173 | Yingtan | SC | 0.1118 |
| 17 | Hebi | NC | 0.1719 | 174 | Xinyu | SC | 0.2077 |
| 18 | Changsha | SC | 0.0307 | 175 | Pingxiang | SC | 0.0289 |
| 19 | Zhuzhou | SC | 0.0872 | 176 | Lanzhou | NC | 1.0730 |
| 20 | Hengyang | SC | 0.2886 | 177 | Jiuquan | NWC | 0.7433 |
| 21 | Yueyang | SC | 0.0508 | 178 | Tianshui | NC | 1.4844 |
| 22 | Xiangtan | SC | 0.0997 | 179 | Jiayuguan | NWC | 0.3389 |
| 23 | Changde | SC | 0.1155 | 180 | Baiyin | NC | 0.5266 |
| 24 | Chenzhou | SC | 0.0738 | 181 | Dingxi | NC | 0.7775 |
| 25 | Shaoyang | SC | 0.0892 | 182 | Pingliang | NC | 1.6761 |
| 26 | Huaihua | SC | 0.0935 | 183 | Weiwu | NWC | 0.5208 |
| 27 | Loudi | SC | 0.2808 | 184 | Jinchang | NWC | 1.1115 |
| 28 | Yongzhou | SC | 0.0653 | 185 | Longnan | SC | 0.7098 |
| 29 | Yiyang | SC | 0.0931 | 186 | Linxia | NC | 0.5443 |
| 30 | Xiangxi | SC | 0.1283 | 187 | Ganann | QT | 0.4424 |
| 31 | Zhangjiajie | SC | 0.2134 | 188 | Lijiang | SC | 0.0397 |
| 32 | Changchun | NC | 0.2007 | 189 | Honghe | SC | 0.0189 |
| 33 | Jilin | NC | 0.1146 | 190 | Qujing | SC | 0.0335 |
| 34 | Yanbian | NC | 0.0743 | 191 | Xishuangbanna | SC | 0.3269 |
| 35 | Siping | NC | 0.3905 | 192 | Yuxi | SC | 0.4144 |
| 36 | Tonghua | NC | 0.1359 | 193 | Liangshan | SC | 0.1730 |
| 37 | Songyuan | NC | 0.1609 | 194 | Panzhihua | SC | 0.4385 |
| 38 | Xing’anmeng | NWC | 0.2357 | 195 | Dehong | SC | 0.0275 |
| 39 | Baishan | NC | 0.1004 | 196 | Baoshan | SC | 0.0701 |
| 40 | Liaoyuan | NC | 0.2405 | 197 | Pu’er | SC | 0.0330 |
| 41 | Yinchuan | NWC | 1.4373 | 198 | Lincang | SC | 0.0134 |

| **Table S1** (continued) | | | | | | | |
| --- | --- | --- | --- | --- | --- | --- | --- |
| Sample number | City’s name | Region | Sr conc^a^  (mg/L) | Sample number | City’s name | Region | Sr conc^a^  (mg/L) |
| 42 | Yulin | NC | 0.4702 | 199 | Panjin | NC | 0.2831 |
| 43 | Wuhai | NWC | 0.9351 | 200 | Jinzhou | NC | 0.4038 |
| 44 | Shizuishan | NWC | 0.8633 | 201 | Chifeng | NWC | 0.4541 |
| 45 | Wuzhong | NWC | 0.5968 | 202 | Huludao | NC | 0.2667 |
| 46 | Zhongwei | NWC | 0.6713 | 203 | Fuxin | NC | 0.4344 |
| 47 | Guyuan | NC | 2.0215 | 204 | Chaoyang | NC | 0.8214 |
| 48 | Yantai | NC | 0.6659 | 205 | Linyi | NC | 0.4252 |
| 49 | Weihai | NC | 0.6177 | 206 | Jining | NC | 0.6089 |
| 50 | Xuzhou | NC | 0.6285 | 207 | Heze | NC | 1.3135 |
| 51 | Yancheng | SC | 0.2742 | 208 | Zaozhuang | NC | 0.5265 |
| 52 | Huai’an | SC | 0.2870 | 209 | Jinan | NC | 0.3651 |
| 53 | Lianyungang | NC | 0.3041 | 210 | Zibo | NC | 0.4893 |
| 54 | Suqian | NC | 0.2801 | 211 | Tai’an | NC | 0.1876 |
| 55 | Urumchi | NWC | 1.1309 | 212 | Dezhou | NC | 0.8559 |
| 56 | Karamay | NWC | 0.2007 | 213 | Binzhou | NC | 0.8741 |
| 57 | Bayingolin | NWC | 0.5259 | 214 | Laiwu | NC | 0.2869 |
| 58 | Kekedala | NWC | 1.0628 | 215 | Hefei | SC | 0.1074 |
| 59 | Yining | NWC | 0.4992 | 216 | Wuhu | SC | 0.2495 |
| 60 | Changji | NWC | 0.5040 | 217 | Bengbu | SC | 0.2964 |
| 61 | Hami | NWC | 0.4667 | 218 | Maanshan | SC | 0.1605 |
| 62 | Turpan | NWC | 0.3100 | 219 | Chuzhou | SC | 0.1516 |
| 63 | Bortala | NWC | 0.7843 | 220 | Anqing | SC | 0.2673 |
| 64 | Hotan | NWC | 0.7865 | 221 | Liu’an | SC | 0.1193 |
| 65 | Xiangyang | SC | 0.1624 | 222 | Fuyang | NC | 0.1213 |
| 66 | Shiyan | SC | 0.1880 | 223 | Xuancheng | SC | 0.1095 |
| 67 | Jingmen | SC | 0.2791 | 224 | Huangshan | SC | 0.0595 |
| 68 | Suizhou | SC | 0.1706 | 225 | Huainan | SC | 0.3455 |
| 69 | Xining | QT | 0.2559 | 226 | Suzhou | NC | 0.8480 |
| 70 | Lhasa | QT | 0.1403 | 227 | Tongling | SC | 0.2414 |
| 71 | Haixi | QT | 0.8200 | 228 | Huaibei | NC | 0.9898 |
| 72 | Haidong | QT | 0.2673 | 229 | Bozhou | NC | 0.7347 |
| 73 | Hainan | QT | 0.9205 | 230 | Chizhou | SC | 0.0396 |
| 74 | Haibei | QT | 0.3420 | 231 | Hangzhou | SC | 0.0449 |
| 75 | Yushu | QT | 0.3454 | 232 | Jiaxing | SC | 0.1886 |
| 76 | Huangnan | QT | 1.0811 | 233 | Jinhua | SC | 0.0204 |
| 77 | Guoluo | QT | 0.2733 | 234 | Huzhou | SC | 0.2250 |
| 78 | Xi’an | NC | 0.1072 | 235 | Quzhou | SC | 0.0125 |
| 79 | Xianyang | NC | 1.0225 | 236 | Haikou | SC | 0.8371 |
| 80 | Weinan | NC | 0.9651 | 237 | Sanya | SC | 0.0719 |
| 81 | Baoji | NC | 0.7948 | 238 | Zhanjiang | SC | 0.0591 |
| 82 | Hanzhong | SC | 0.3400 | 239 | Maoming | SC | 0.0323 |

| **Table S1** (continued) | | | | | | | |
| --- | --- | --- | --- | --- | --- | --- | --- |
| Sample number | City’s name | Region | Sr conc^a^  (mg/L) | Sample number | City’s name | Region | Sr conc^a^  (mg/L) |
| 83 | Yan’an | NC | 0.8191 | 240 | Yangjiang | SC | 0.0483 |
| 84 | Ankang | SC | 0.1970 | 241 | Danzhou | SC | 0.0490 |
| 85 | Tongchuan | NC | 0.9622 | 242 | Haerbin | NC | 0.0737 |
| 86 | Qingyang | NC | 3.1131 | 243 | Daqing | NC | 0.5063 |
| 87 | Wuhan | SC | 0.2697 | 244 | Qiqihaer | NC | 0.3695 |
| 88 | Yichang | SC | 0.2977 | 245 | Mudanjiang | NC | 0.1232 |
| 89 | Jingzhou | SC | 0.3040 | 246 | Jiamusi | NC | 0.1428 |
| 90 | Xiaogan | SC | 0.1703 | 247 | Hulunbeier | NWC | 0.1254 |
| 91 | Huanggang | SC | 0.2691 | 248 | Suihua | NC | 0.3457 |
| 92 | Huangshi | SC | 0.2645 | 249 | Jixi | NC | 0.1062 |
| 93 | Xianning | SC | 0.1972 | 250 | Shuangyashan | NC | 0.9255 |
| 94 | Enshi | SC | 0.5784 | 251 | Heihe | NC | 0.0843 |
| 95 | Ezhou | SC | 0.2943 | 252 | Hegang | NC | 0.0509 |
| 96 | Suzhou | SC | 0.2138 | 253 | Daxinganling | NC | 0.1246 |
| 97 | Wuxi | SC | 0.2258 | 254 | Qitaihe | NC | 0.1497 |
| 98 | Changzhou | SC | 0.2509 | 255 | Guiyang | SC | 0.5900 |
| 99 | Nantong | SC | 0.2458 | 256 | Zunyi | SC | 0.1442 |
| 100 | Tianjin | NC | 0.1639 | 257 | Qiandongnan | SC | 0.0619 |
| 101 | Cangzhou | NC | 0.3590 | 258 | Qiannan | SC | 0.1031 |
| 102 | Tangshan | NC | 0.4263 | 259 | Anshun | SC | 0.5163 |
| 103 | Qinhuangdao | NC | 0.3160 | 260 | Qianxinan | SC | 0.2658 |
| 104 | Taiyuan | NC | 0.5139 | 261 | Bijie | SC | 1.5935 |
| 105 | Jinzhong | NC | 1.3414 | 262 | Liupanshui | SC | 0.4383 |
| 106 | Datong | NC | 1.5572 | 263 | Tongren | SC | 0.0773 |
| 107 | Yuncheng | NC | 0.7490 | 264 | Guangzhou | SC | 0.1269 |
| 108 | Linfen | NC | 0.9656 | 265 | Dongguan | SC | 0.0616 |
| 109 | Changzhi | NC | 0.6005 | 266 | Foshan | SC | 0.0901 |
| 110 | Xinzhou | NC | 0.1880 | 267 | Zhuhai | SC | 0.2216 |
| 111 | Lvliang | NC | 0.3073 | 268 | Huizhou | SC | 0.0307 |
| 112 | Jincheng | NC | 1.4737 | 269 | Jiangmen | SC | 0.1596 |
| 113 | Yangquan | NC | 1.5949 | 270 | Shantou | SC | 0.0645 |
| 114 | Shuozhou | NC | 0.4166 | 271 | Zhaoqing | SC | 0.1603 |
| 115 | Shijiahzuang | NC | 0.3203 | 272 | Jieyang | SC | 0.0084 |
| 116 | Baoding | NC | 0.1917 | 273 | Qingyuan | SC | 0.0673 |
| 117 | Handan | NC | 0.1720 | 274 | Shaoguan | SC | 0.1300 |
| 118 | Xingtai | NC | 0.1745 | 275 | Heyuan | SC | 0.0248 |
| 119 | Hengshui | NC | 0.2373 | 276 | Zhongshan | SC | 0.1551 |
| 120 | Shenyang | NC | 0.4553 | 277 | Yunfu | SC | 0.0322 |
| 121 | Anshan | NC | 0.2006 | 278 | Fuzhou | SC | 0.0550 |
| 122 | Fushun | NC | 0.2097 | 279 | Putian | SC | 0.0507 |
| 123 | Liaoyang | NC | 0.1958 | 280 | Ningde | SC | 0.0291 |

| **Table S1** (continued) | | | | | | | |
| --- | --- | --- | --- | --- | --- | --- | --- |
| Sample number | City’s name | Region | Sr conc^a^  (mg/L) | Sample number | City’s name | Region | Sr conc^a^  (mg/L) |
| 124 | Benxi | NC | 0.1731 | 281 | Nanping | SC | 0.0731 |
| 125 | Tongliao | NC | 0.4773 | 282 | Sanming | SC | 0.0314 |
| 126 | Tieling | NC | 0.2121 | 283 | Dalian | NC | 0.1830 |
| 127 | Shenzhen | SC | 0.0350 | 284 | Dandong | NC | 0.1161 |
| 128 | Shanghai | SC | 0.2128 | 285 | Yingkou | NC | 0.1174 |
| 129 | Xiamen | SC | 0.1266 | 286 | Chengdu | SC | 0.2415 |
| 130 | Quanzhou | SC | 0.0370 | 287 | Mianyang | SC | 0.2876 |
| 131 | Zhangzhou | SC | 0.1088 | 288 | Leshan | SC | 0.2963 |
| 132 | Longyan | SC | 0.1970 | 289 | Deyang | SC | 0.3495 |
| 133 | Qingdao | NC | 0.7028 | 290 | Meishan | SC | 0.2291 |
| 134 | Weifang | NC | 0.2898 | 291 | Neijiang | SC | 0.3663 |
| 135 | Rizhao | NC | 0.2299 | 292 | Suining | SC | 0.3266 |
| 136 | Ningbo | SC | 0.0361 | 293 | Ya’an | SC | 0.2872 |
| 137 | Wenzhou | SC | 0.0301 | 294 | Ziyang | SC | 0.2684 |
| 138 | Shaoxing | SC | 0.0629 | 295 | Zigong | SC | 0.2960 |
| 139 | Hezhou | SC | 0.0319 | 296 | Guangyuan | SC | 0.2900 |
| 140 | Zhoushan | SC | 0.0772 | 297 | Aba | QT | 0.2871 |
| 141 | Lishui | SC | 0.0454 | 298 | Nanchong | SC | 0.4053 |
| 142 | Hohhot | NWC | 0.3586 | 299 | Ganzi | QT | 0.0942 |
| 143 | Baotou | NWC | 1.0151 | 300 | Beijing | NC | 0.6935 |
| 144 | Erdos | NWC | 0.6307 | 301 | Zhangjiakou | NC | 0.9803 |
| 145 | Ulanqab | NWC | 0.7738 | 302 | Chengde | NC | 0.9541 |
| 146 | Xilin Gol | NWC | 0.6385 | 303 | Taizhou | SC | 0.2328 |
| 147 | Bayannur | NWC | 0.9611 | 304 | Hancheng | NC | 0.6054 |
| 148 | Nanning | SC | 0.0708 | 305 | Dongying | NC | 0.9606 |
| 149 | Guilin | SC | 0.0280 | 306 | Aksu | NWC | 1.0129 |
| 150 | Liuzhou | SC | 0.0649 | 307 | Tacheng | NWC | 0.9357 |
| 151 | Yulin | SC | 0.1222 | 308 | Zhaotong | SC | 0.0869 |
| 152 | Beihai | SC | 0.0104 | 309 | Chuxiong | SC | 0.3631 |
| 153 | Wuzhou | SC | 0.1138 | 310 | Dongfang | SC | 0.0972 |
| 154 | Baise | SC | 0.0683 | 311 | Wenshan | SC | 0.0567 |
| 155 | Qinzhou | SC | 0.0792 | 312 | Altay | NWC | 0.0414 |
| 156 | Guigang | SC | 0.0743 | 313 | Diqing | SC | 0.1310 |
| 157 | Hechi | SC | 0.0917 | 314 | Dali | SC | 0.1572 |

^a^ Sr conc is short for Sr concentraion

**Table S2** Prevalence rate of rickets in different cities in China

| City | Sample number | Year | Prevalence (%) | Sr conc^a^  (mg/L) | Ca/Sr ratio  (in mg/L) | Refer-ences |
| --- | --- | --- | --- | --- | --- | --- |
| Xi’an | 3085 | 2014-2017 | 38.2 | 0.2559 | 187.7 | [1] |
| Shanghai | 769 | 2005 | 22.4 | 0.2128 | 174.3 | [2] |
| Suzhou | 326 | 2011 | 13.6 | 0.2138 | 178.7 | [3] |
| Yangquan | 6334 | 2005-2008 | 30.9 | 1.5949 | 51.0 | [4] |
| Quanzhou | 3065 | 2001-2009 | 21.4 | 0.0370 | 125.4 | [5] |
| Urumqi | 880 | 1990-1995 | 43.6 | 1.1309 | 125.4 | [6] |
| Beihai | 386 | 1999-2001 | 11.1 | 0.0104 | 91.7 | [7] |
| Jixi | 21000 | 2010 | 30.6 | 0.1062 | 1107.6 | [8] |
| Tianjing | 42260 | 2004 | 5.7 | 0.1639 | 163.2 | [9] |
| Taiyuan | 844 | 2008 | 35.8 | 0.5139 | 77.0 | [10] |
| Hinggan league | 1072 | 2008 | 38.5 | 0.2357 | 172.2 | [11] |
| Guangzhou | 1211 | 2008 | 14.5 | 0.1269 | 153.8 | [12] |
| Zhongwei | 1674 | 2012 | 41.8 | 0.6713 | 201.5 | [13] |
| Xi’an | 651 | 2009-2012 | 13.2 | 0.1072 | 118.8 | [14] |
| Shuangyashan | 1984 | 2004 | 42.1 | 0.9255 | 102.4 | [15] |
| Jiamusi | 3638 | 2001 | 41.1 | 0.1428 | 156.0 | [16] |
| Enshi | 1858 | 2005 | 20.0 | 0.5784 | 161.9 | [17] |
| Shenyang | 483 | 2015-2018 | 23.8 | 0.4553 | 181.9 | [18] |
| Zunyi | 10618 | 2000-2005 | 31.0 | 0.1442 | 251.0 | [19] |
| Shenzhen | —— | 2017 | 0.4 | 0.0350 | 144.4 | [20] |
| Lasa | 1311 | 2012 | 26.0 | 0.1403 | 57.8 | [21] |
| Wuhan | 719 | 2003 | 16.9 | 0.2697 | 235.5 | [22] |
| Xuancheng | 552 | 2016 | 30.0 | 0.1095 | 140.1 | [23] |
| Leshan | 2400 | 2005 | 27.4 | 0.2963 | 227.9 | [24] |
| Chengdou | 680 | 2009-2010 | 22.1 | 0.2415 | 282.2 | [25] |
| Jincheng | 1624 | 1999 | 47.7 | 1.4737 | 1106.6 | [26] |
| Changchun | 1391 | 2008 | 44.5 | 0.2007 | 134.2 | [27] |
| Tangshan | 5009 | 2007 | 41.8 | 0.4263 | 98.1 | [28] |
| Changzhou | 965 | 2009 | 41.1 | 0.2509 | 239.1 | [29] |
| Shaoxin | 5004 | 2005 | **7.2** | 0.0629 | 85.3 | [30] |
| Sanming | 2758 | 2010 | 25.2 | 0.0314 | 68.8 | [31] |
| Jiujiang | 842 | 2004 | 49.6 | 0.2923 | 140.5 | [32] |
| Dongying | 526 | 2005 | 39.2 | 0.9606 | 222.3 | [33] |
| Nanyang | 6119 | 2010 | 42.2 | 0.1462 | 139.3 | [34] |
| Ezhou | 663 | 2008 | 23.4 | 0.2943 | 735.4 | [35] |
| Changsha | 806 | 2010 | 41.7 | 0.0307 | 139.7 | [36] |
| Liuzhou | 980 | 2008 | 22.7 | 0.0649 | 143.3 | [37] |
| Chongqing | 3034 | 2007 | 11.6 | 0.3794 | 88.6 | [38] |
| Aba | 664 | 2005 | 34.0 | 0.2871 | 175.0 | [39] |
| Qiannan | 2115 | 2005 | 30.0 | 0.1031 | 239.8 | [40] |
| Lanzhou | 19822 | 2000 | 21.4 | 1.0730 | 283.5 | [41] |
| Karamay | 638 | 2003 | 58.9 | 0.2007 | 159.5 | [42] |
| **Table S2** (continued) | | | | | | |
| City | N | Year | Prevalence (%) | Sr conc^a^  (mg/L) | Ca/Sr ratio  (in mg/L) | Refer-ences |
| Yingkou | 1345 | 2013~2015 | 27.8 | 0.1174 | 255.9 | [43] |
| Nanning | 3678 | 2011 | 27.3 | 0.0708 | 146.9 | [44] |
| Hukou | 7894 | 1996 | 19.6 | 0.2250 | 279.6 | [45] |
| Zhaoqing | 700 | 2003 | 34.3 | 0.1603 | 240.2 | [46] |
| Haikou | 804 | 2004 | 60.5 | 0.8371 | 23.3 | [47] |
| Guilin | 1960 | 2003-2004 | 13.6 | 0.0280 | 577.1 | [48] |

^a^ Sr conc is short for Sr concentraion **Table S3** Bone mineral density (BMD) of 60-70 years old residents in 31 cities in China

| City | Sample number | Year | Lumbar BMD  (g/cm^3^) | | Femoral neck BMD (g/cm^3^) | | Ca conc^a^  (mg/L) | Sr conc^a^  (mg/L) | Refer-  ence |
| --- | --- | --- | --- | --- | --- | --- | --- | --- | --- |
|  |  |  | Male | Female | Male | Female |  |  |  |
| Shenyang | 1826 | 2014-2017 | 1.03 | 0.899 | 0.839 | 0.679 | 79.6680 | 0.4553 | [49] |
| Lanzhou | 1212 | 2016-2018 | 1.084 | 0.927 | 0.830 | 0.862 | 91.5420 | 1.0730 | [50] |
| Liuzhou | 1230 | 2006 |  |  | 0.768 | 0.729 | 71.7970 | 0.0649 | [51] |
| Chengdu | 1196 | 2000 | 1.007 | 0.876 | 0.805 | 0.711 | 39.4190 | 0.2415 | [52] |
| Harbin | 759 | 2015 | 0.990 | 0.820 |  |  | 10.0740 | 0.0737 | [53] |
| Beijing | 3859 | 2017-2018 | 1.19 | 1.01 | 0.92 | 0.81 | 43.7670 | 0.6935 | [54] |
| Dalian | 1112 | 2002 |  |  | 0.751 | 0.740 | 24.2190 | 0.1830 | [55] |
| Daqing | 1096 | 2008-2010 | 1.104 | 0.950 | 0.846 | 0.773 | 39.0660 | 0.5063 | [56] |
| Foshan | 839 | 2006-2008 | 0.903 | 0.723 | 0.909 | 0.768 | 32.0000 | 0.0901 | [57] |
| Longyan | 1989 | 2004 | 1.043 | 0.809 | 0.770 | 0.702 | 63.6710 | 0.1970 | [58] |
| Jiangmen | 2454 | 1995-2005 | 0.92 | 1 | 0.85 | 0.81 | 46.2640 | 0.1596 | [59] |
| Hezhou | 3000 | 2009 | 0.858 | 0.751 | 0.708 | 0.658 | 30.6210 | 0.0475 | [60] |
| Guangzhou | 2103 | 2003-2010 | 0.82 | 0.72 | 0.79 | 0.71 | 35.8120 | 0.1269 | [61] |
| Haikou | 2176 | 2020 | 1.02 | 0.80 | 0.84 | 0.70 | 19.5380 | 0.8371 | [62] |
| Hohhot | 1000 | 2009 | 0.93 | 0.83 |  |  | 47.8620 | 0.3586 | [63] |
| Huizhou | 896 | 2011-2012 | 0.886 | 0.772 |  |  | 8.5507 | 0.0307 | [64] |
| Jinan | 411 | 2005-2008 | 1.113 | 0.944 | 0.909 | 0.764 | 121.4600 | 0.3651 | [65] |
| Luzhou | 375 | 2015 |  |  |  | 0.755 | 36.5710 | 0.2441 | [66] |
| Mianyang | 5039 | 2016 | 0.893 | 0.893 | 0.807 | 0.807 | 41.4920 | 0.2876 | [67] |
| Nanchang | 548 | 2012 | 0.882 |  | 0.930 |  | 19.7020 | 0.0774 | [68] |
| Nanjing | 5168 | 2002 | 1.075 | 0.900 | 0.862 | 0.719 | 38.5290 | 0.2331 | [69] |
| Yueyang | 355 | 2011-2012 | 0.762 | 0.674 | 0.735 | 0.651 | 7.3961 | 0.0508 | [70] |
| Qingdao | 4434 | 2008-2010 | 1.07 | 0.900 | 0.861 | 0.720 | 39.0870 | 0.7028 | [71] |
| Xiamen | 6233 | 2007-2010 | 0.929 | 0.899 | 0.839 | 0.786 | 20.2560 | 0.1266 | [72] |
| Shanghai | 884 | 2008-2009 |  | 0.924 |  | 0.755 | 37.0930 | 0.2128 | [73] |
| Shenzhen | 747 | 2000-2003 | 0.883 | 0.770 | 0.826 | 0.713 | 9.9111 | 0.0350 | [74] |
| Wuxi | 1582 | 2002 | 0.904 | 0.746 | 0.792 | 0.703 | 39.7840 | 0.2258 | [75] |
| **Table S3** (continued) | | | | | | | | | |
| City | Sample number | Year | Lumbar BMD  (g/cm^3^) | | Femora neck BMD (g/cm^3^) | | Ca conc^a^  (mg/L) | Sr conc^a^  (mg/L) | Refer-  ence |
|  |  |  | Male | Female | Male | Female |  |  |  |
| Xi'an | 2380 | 2003-2008 | 0.930 | 0.834 | 0.814 | 0.671 | 23.8230 | 0.1072 | [76] |
| Xining | 1244 | 2011 | 1.106 | 0.867 |  |  | 48.0260 | 0.2559 | [77] |
| Yan'an | 2416 | 2009-2011 | 0.921 | 0.773 | 0.809 | 0.699 | 43.9840 | 0.8191 | [78] |
| Xiongchu | 120 | 2013 | 0.965 | 0.696 |  |  | 40.4330 | 0.3631 | [79] |

^a^ Sr conc is short for Sr concentraion

**Table S4.** Health risks of Sr in public drinking water in Chinese cities (mg/L).

| Age | Statistical indicator | Overall | | | |  | SC | | |  | NC | | |  | QN | | |
| --- | --- | --- | --- | --- | --- | --- | --- | --- | --- | --- | --- | --- | --- | --- | --- | --- | --- |
| groups |  | HQ_i_^a^ | | HQ_d_^b^ | HI^c^ |  | HQ_i_ | HQ_d_ | HI |  | HQ_i_ | HQ_d_ | HI |  | HQ_i_ | HQ_d_ | HI |
| Infants | Mean | 0.0653 | 0.0010 | | 0.0663 |  | 0.0308 | 0.0005 | 0.0313 |  | 0.0924 | 0.0015 | 0.0938 |  | 0.1028 | 0.0017 | 0.1045 |
|  | Mid | 0.0289 | 0.0001 | | 0.0297 |  | 0.0169 | 0 | 0.0170 |  | 0.0534 | 0.0001 | 0.0541 |  | 0.0762 | 0.0002 | 0.0772 |
|  | Max | 1.0181 | 0.0914 | | 1.0202 |  | 0.2769 | 0.0543 | 0.2774 |  | 0.7938 | 0.1641 | 0.7954 |  | 0.4685 | 0.2272 | 0.4688 |
|  | 95%^d^ | 0.2469 | 0.0031 | | 0.2469 |  | 0.1057 | 0.0016 | 0.1090 |  | 0.3061 | 0.0049 | 0.0938 |  | 0.2798 | 0.0054 | 0.2860 |
|  | SD | 0.1053 | 0.0048 | | 0.1064 |  | 0.0375 | 0.0026 | 0.0381 |  | 0.1074 | 0.0079 | 0.1091 |  | 0.0908 | 0.0101 | 0.0930 |
| Children | Mean | 0.0404 | 0.0003 | | 0.0408 |  | 0.0189 | 0.0002 | 0.0191 |  | 0.0567 | 0.0005 | 0.0572 |  | 0.0627 | 0.0006 | 0.0633 |
|  | Mid | 0.0158 | 0 | | 0.0161 |  | 0.0090 | 0 | 0.0090 |  | 0.0291 | 0.0001 | 0.0293 |  | 0.0401 | 0.0001 | 0.0403 |
|  | Max | 0.7741 | 0.0326 | | 0.7749 |  | 0.2007 | 0.0194 | 0.2009 |  | 0.5752 | 0.0585 | 0.5758 |  | 0.4070 | 0.0810 | 0.4074 |
|  | 95% | 0.1480 | 0.0011 | | 0.1491 |  | 0.0674 | 0.0006 | 0.0684 |  | 0.1969 | 0.0017 | 0.1988 |  | 0.1973 | 0.0019 | 0.1980 |
|  | SD | 0.0726 | 0.0017 | | 0.0729 |  | 0.0259 | 0.0009 | 0.0261 |  | 0.0746 | 0.0028 | 0.0751 |  | 0.0645 | 0.0036 | 0.0652 |
| Teens | Mean | 0.0184 | 0.0002 | | 0.0186 |  | 0.0086 | 0.0001 | 0.0087 |  | 0.0258 | 0.0003 | 0.0261 |  | 0.0285 | 0.0003 | 0.0289 |
|  | Mid | 0.0074 | 0 | | 0.0076 |  | 0.0042 | 0 | 0.0042 |  | 0.0133 | 0 | 0.0135 |  | 0.0186 | 0 | 0.0189 |
|  | Max | 0.3832 | 0.0149 | | 0.3838 |  | 0.0991 | 0.0089 | 0.0992 |  | 0.2839 | 0.0268 | 0.2844 |  | 0.1713 | 0.0371 | 0.1716 |
|  | 95% | 0.0659 | 0.0007 | | 0.0659 |  | 0.0311 | 0.0003 | 0.0314 |  | 0.0901 | 0.0010 | 0.0908 |  | 0.0889 | 0.0011 | 0.0910 |
|  | SD | 0.0328 | 0.0009 | | 0.0330 |  | 0.0117 | 0.0117 | 0.0118 |  | 0.0336 | 0.0014 | 0.0339 |  | 0.0289 | 0.0017 | 0.0293 |
| Adults | Mean | 0.0210 | 0.0002 | | 0.0213 |  | 0.0099 | 0.0001 | 0.0100 |  | 0.0296 | 0.0003 | 0.0299 |  | 0.0500 | 0.0006 | 0.0505 |
|  | Mid | 0.0090 | 0 | | 0.0092 |  | 0.0050 | 0 | 0.0051 |  | 0.0163 | 0 | 0.0164 |  | 0.0422 | 0.0001 | 0.0424 |
|  | Max | 0.3923 | 0.0175 | | 0.3929 |  | 0.1014 | 0.0105 | 0.1016 |  | 0.2907 | 0.0316 | 0.2912 |  | 0.2052 | 0.0485 | 0.2056 |
|  | 95% | 0.0745 | 0.0007 | | 0.0746 |  | 0.0352 | 0.0004 | 0.0359 |  | 0.1021 | 0.0011 | 0.1044 |  | 0.1196 | 0.0020 | 0.1217 |
|  | SD | 0.0360 | 0.0010 | | 0.0363 |  | 0.0128 | 0.0005 | 0.0129 |  | 0.0368 | 0.0016 | 0.0371 |  | 0.0354 | 0.0024 | 0.0359 |

^a^ The ingestion hazard quotient.  ^b^ The dermal hazard quotient.  ^c^ The total health risk via multiple exposure pathways. ^d^ The 95th percentile value

**Table S5.** Correlation between Sr and Ca, Sr and Mg as well as Sr and the total hardness (TH)

| Element | Ca | Mg | TH |
| --- | --- | --- | --- |
| Sr | 0.804^**^ | 0.863^**^ | 0.868 |

^**^ significantly correlated at 0.01 level of probability

**Table S6.** Distributions of strontium concentrations in public drinking water in Chinese cities (mg/L).

| Sampling zone^a^ | N^b^ | Distribution^c^ | AD test^d^ | p-value^e^ | Rank |
| --- | --- | --- | --- | --- | --- |
| NC | 108 | Gamma (0.0503, 0.44, 1.10704) | 0.4958 | 0.315 | 1 |
| SC | 163 | Gamma (0.0050, 0.1542, 1.12985) | 0.8236 | 0.059 | 1 |
| QN | 43 | Beta (0.0181, 1.4764, 1.39693, 2.08438) | 0.2259 | --- | 1 |
| Overall | 314 | Lognormal (0.3802, 0.5382) | 0.7785 | 0.024 | 1 |

^a^ NC: Northern China; SC: Southern China; QN: Qinghai-Tibet Plateau and Northwest China.

^b^ Number of samples.

^c^ The values in the parenthesis are the location parameter, scale parameter, and shape parameter for Gamma distribution; There are minimum value, maximum value, Alpha value, and Beta value in the parenthesis for Beta distribution; The values in parentheses represent the geometric mean and geometric standard deviation for lognormal distribution.

^d^ Anderson-Darling test.

^e^ Significance level


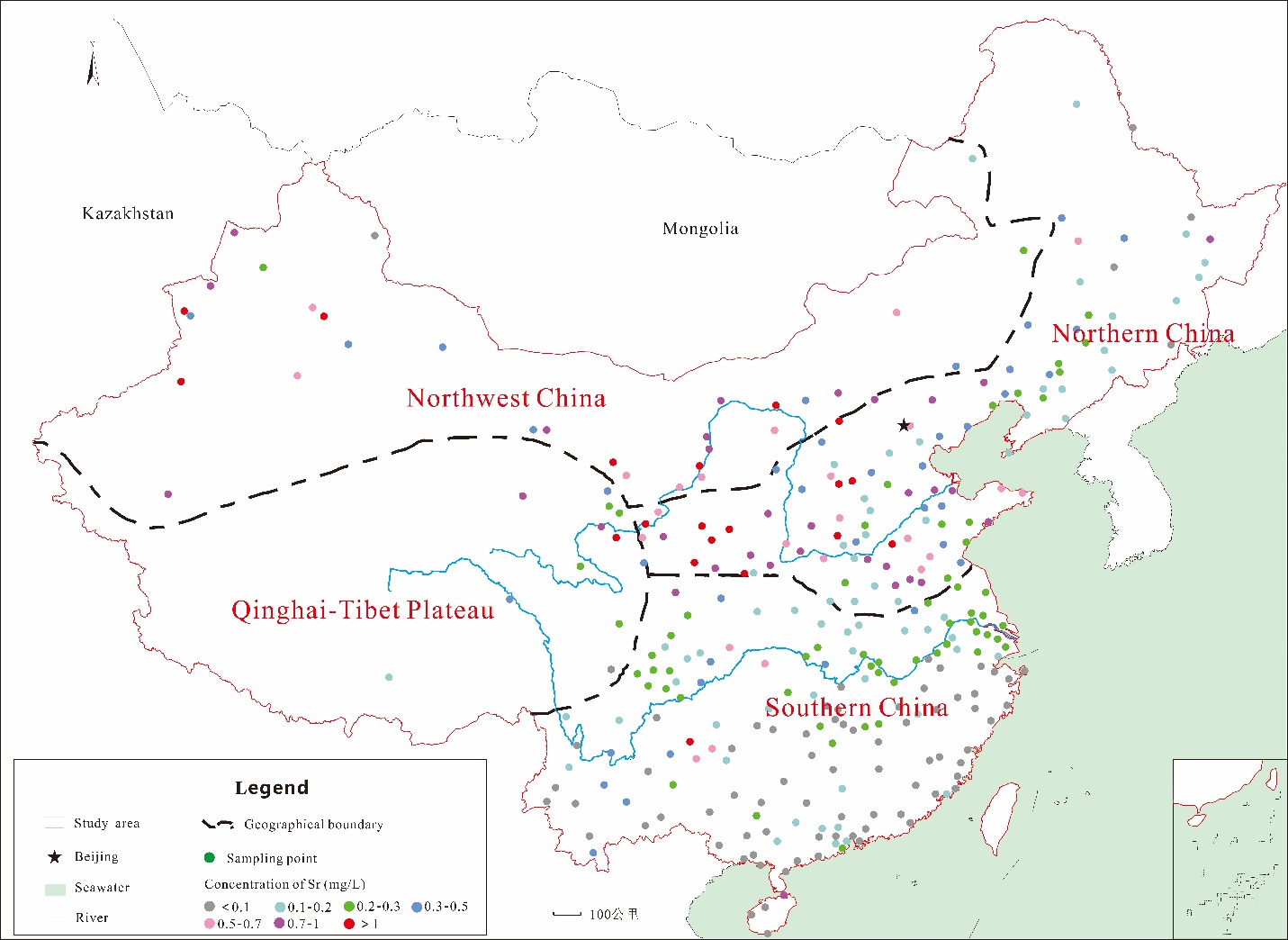


**Fig. S1.** Sample site distribution and strontium concentrations in public drinking water in China.


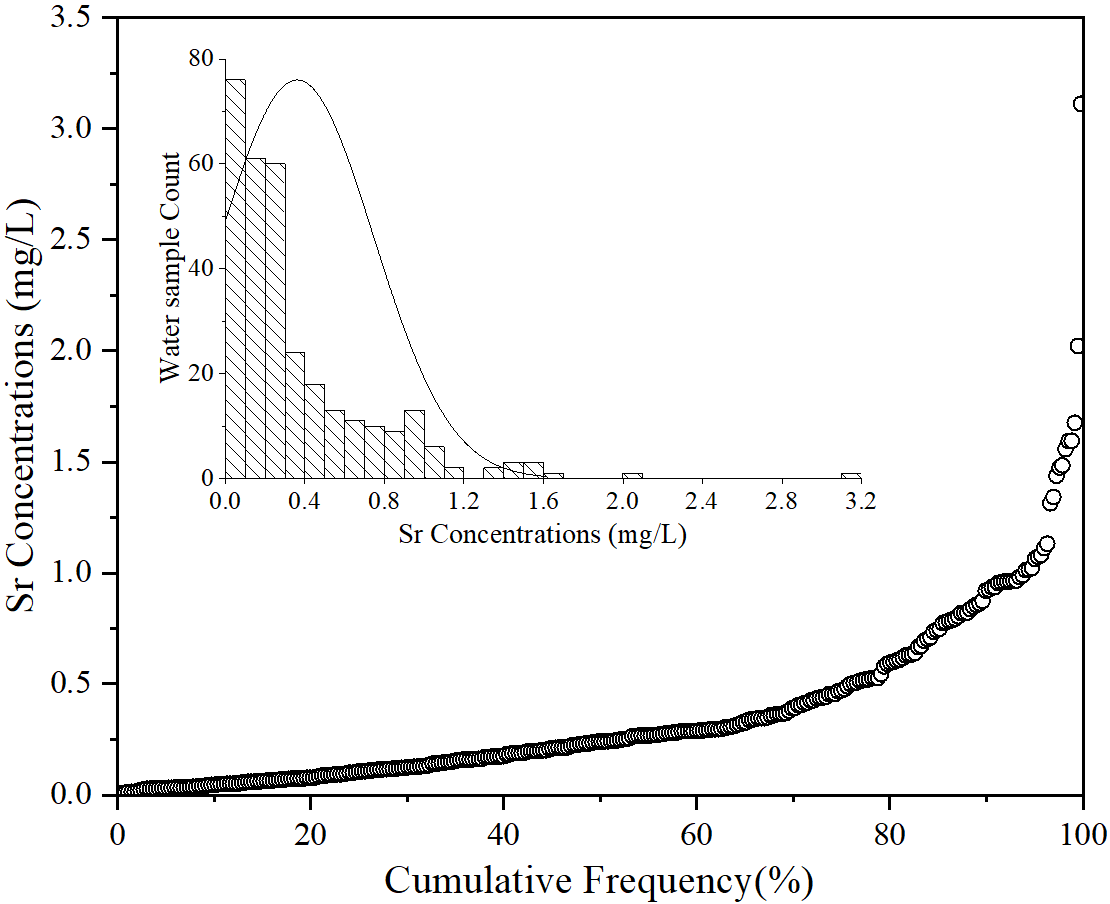


**Fig. S2.** The probability distribution of strontium concentration in public drinking water in China.


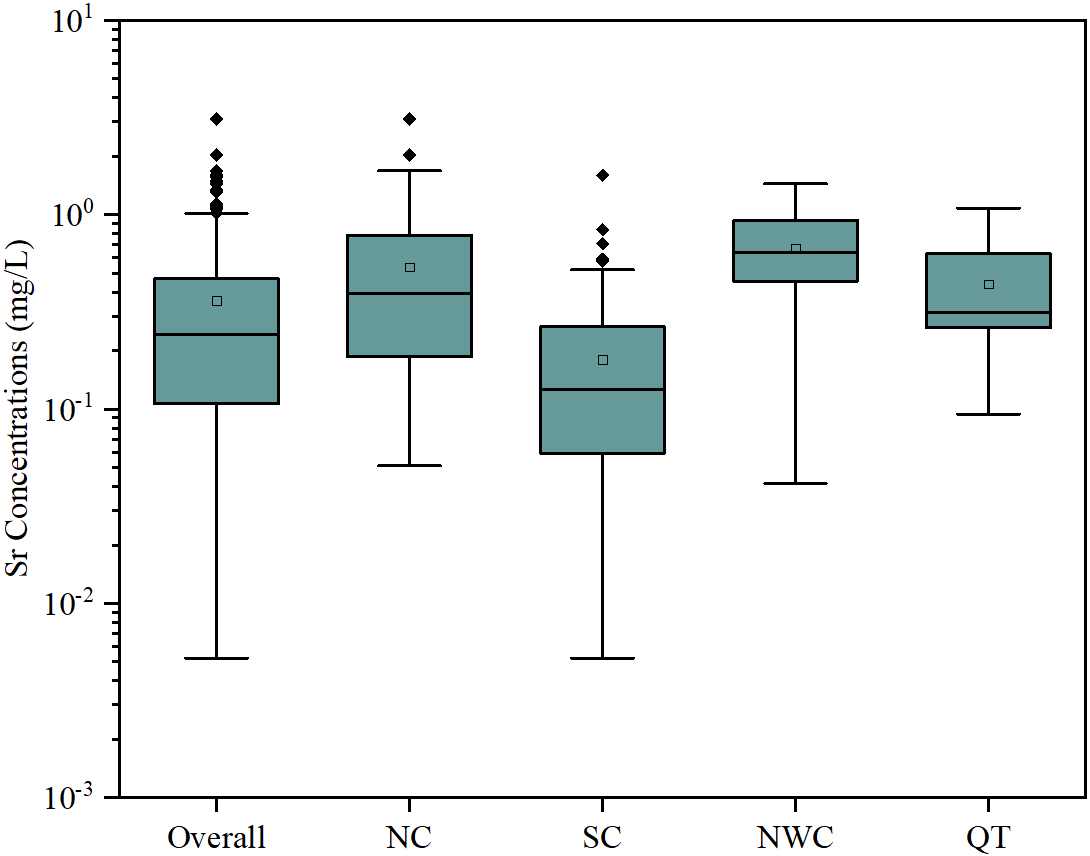


**Fig. S3.** Box diagram showing the concentrations of strontium in water samples from different regions. (NC: Northern China; SC: Southern China; NWC: Northwest China; QT: Qinghai-Tibet Plateau)

*
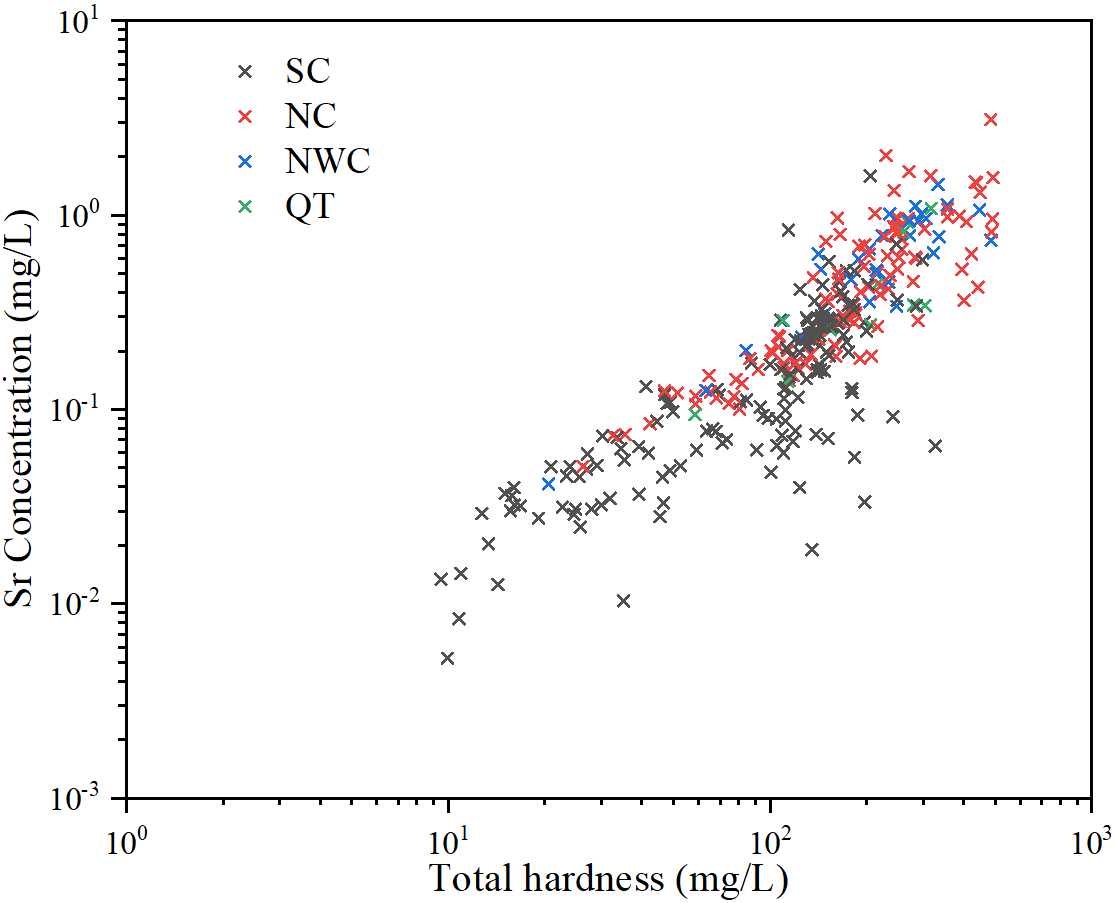
*

**Fig. S4.** The relationship between strontium concentration and total hardness in drinking water samples from different regions. (NC: Northern China; SC: Southern China; NWC: Northwest China; QT: Qinghai-Tibet Plateau)

*
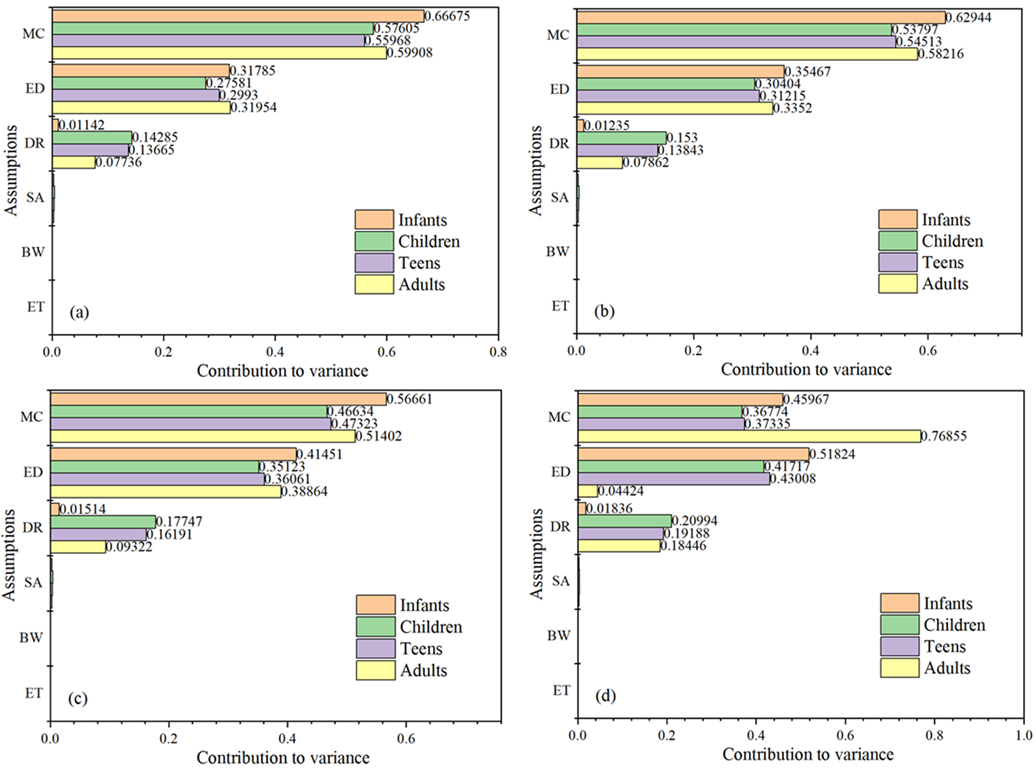
*

**Fig. S5.** Sensitivity analysis of input parameters in four age groups in different regions: (a) Overall; (b) Northern China; (c) Southern China and (d) Qinghai-Tibet Plateau and Northwest China.

**Reference**

1. Ma XQ (2017) Investigation and analysis of rickets in children in Qinghai Province. World Latest Medicine Information (Electronic Version) 78:166 (in Chinese)
2. Gong Q, Yu SM, Zhou XJ (2007) The investigation and analysis orf the status of rickets among children under 6 years of age in shanghai. The 6th National Symposium on rickets prevention and treatment (in Chinese)
3. Han X, Wu Y, Sun CH (2011) Investigation and analysis of rickets in 0-3 years old children in Changshu City. Health industry in China 8(1):99-101(in Chinese)
4. Pan YQ, Li HY, Song ZH (2011) Study of growth and nutritional status of children aged 0-5 years in Yangquan City. Research on maternal and child health in China 22(6):723-726 (in Chinese)
5. Wang YY, Cai LR, Chen LN (2014) Epidemiological investigation of vitamin D deficiency rickets in children under 7 years old in Quanzhou. Pregnancy and child care in China 8(19):119-120 (in Chinese)
6. Zhang W, Wang XZ (1997) Investigation and Analysis on the prevalence of rickets in Jiuyunsi of Urumqi. Journal of Xinjiang medicine 027(002):103-104 (in Chinese)
7. Wu YL (2003) Analysis of the causes of the sudden increase of the incidence of rickets in infants and young children in Beihai City from 2000 to 2001. Guangxi Medical Journal 25(010):2085-2086 (in Chinese)
8. Yu B (2010) Investigation and analysis of rickets in infants and young children in this area. Chinese Medicine Information 2(3): 33-34 (in Chinese)
9. Zhang Y (2006) The Analysis on Outcomes of Physical Examination in Rural Citizens Aged 14 and Below in 2004, Tianjin. Chinese Journal of Prevention and Control of Chronic Non-communicable Diseases 14(5): 317-319 (in Chinese)
10. Wang JQ (2011) Investigation and analysis of children rickets in Ximing mining area. Proceeding of Clinical Medicine 20(4): 289-290 (in Chinese)
11. Yu XL (2010) Investigation and analysis of 1072 cases of rickets in children under 3 years old. Medcial Information 5(12): 3481-3482 (in Chinese)
12. Luo YC (2011) Analysis of the causes of vitamin D deficiency rickets in urban infants. Medcial Information 24(1): 98-100 (in Chinese)
13. Shi JY (2011) Investigation and analysis of rickets of 1674 children under 3 years old. Psychological Doctor 1: 99-100 (in Chinese)
14. Yang L (2013) Analysis of rickets surveillance in children aged 0-3 years in Xidajie District. Nei Mongol Journal of Traditional Chinese Medicine 27:124-125 (in Chinese)
15. Yang L, Zheng DP (2006) Investigation on rickets of 0-3 years old children in Baoqing County. Heilongjiang Medicine and Pharmacy 4:3 (in Chinese)
16. Yuan AM, Wang BL, Lou BL (2003) Investigation on rickets of 0-3 years old children in Jiamusi City. Heilongjiang Medicine and Pharmacy 026:87 (in Chinese)
17. Zhu Q, Su HC, Wang BQ (2007) Analysis and investigation on rachitis in the downtown of Enshi city. Chinese Journal of Traditional Medical Traumatology & Orthopedics 15(012):28-30 (in Chinese)
18. Wang W (2018) Characteristics and influencing factors of rickets of children under 3 years old. China Pharmaceuticals 28(Z1):230-231 (in Chinese)
19. Ouyang X, Ouyang KY (2006) Prevention of rickets of children under 3 years old and health education guidance for parents. Modern Medicine & Health (in Chinese)
20. Li WY, Chen S (2019) Comparison and analysis of health care services and status of 0-3-year-old children in social health center of Pingshan people's Hospital of Shenzhen from 2013 to 2017. Modern Diagnosis and Treatment 030(014):2440-2442 (in Chinese)
21. Zhang L, Da W, Qiao HB (2013) Analysis of physical examination of children aged 0-3 years in Deqing County, Lasa City. Tibetan Medicine 34(1):56-58 (in Chinese)
22. Wang J, Yang ZX, Wang LG (2005) Investigation on growth and development of children aged 0-3 years in community and evaluation of health promotion. China Medical Herald 2(12):129-129 (in Chinese)
23. Wu Y (2018) Prevalence and risk factors of rickets in children in Jingxian, Xuancheng, Anhui Provinc. Maternal and Child Health Care of China 33(15):3514-3517 (in Chinese)
24. Shi LJ (2010) Prevention and treatment of vitamin D deficiency rickets in children aged 0-4 years. Journal of Management scientist 000(001):200-201 (in Chinese)
25. Wang YM, Cao RH, Liu HS (2011) Influencing Factors and Morbidity of Rickets with Nutritional Vitamin D Deficiency in the Children Under 3 Years Old in Taixing Town. Chinese Primary Health Care 11:50-52 (in Chinese)
26. Pang CL, Li Q, Wei HM (2000) Prevalence of rickets in infants aged 0-3 years in Gaoping City. Journal of Changzhi Medical College 04:258-259 (in Chinese)
27. Li XC (2008) Investigation on the incidence of rickets in rural children of Changchun City. Chin Matern Child Health Care 23(30):4367-4368 (in Chinese)
28. Du WR (2012) Relationship between rickets and trace elements in children. Chin Matern Child Health Care 27(2):231-233 (in Chinese)
29. Zeng YS (2009) Investigation of rickets in 965 infants under 5 years old. Harbin Medical Journal 29(6): 60-62 (in Chinese)
30. Cao ZF (2007) Analysis of bone alkaline phosphatase of 5004 children in Zhuji City Management of rural health service in China 27(3):223-224 (in Chinese)
31. Hu GY (2011) Analysis of bone alkaline phosphatase of 2758 children aged 0-3 years in Yong'an City. Modern Journal of Integrated Traditional Chinese and Western Medicine 20(32): 4089-4090 (in Chinese)
32. Hu HD (2007) Research on relationship between rickets and blood lead level in babies and infants.Chin Matern Child Health Care 22(27):3818-3819 (in Chinese)
33. Chen CL (2010) Investigation on vitamin D deficiency rickets in children under 3 years old in a community of Dongying City. Shandong Medical Journal 50(13):62-63 (in Chinese)
34. He F (2010) Epidemiological and etiological investigation of vitamin D deficiency rickets in rural areas of Nanyang. Clinical Focus 25(11):991-993 (in Chinese)
35. Zhang HL (2010) Investigation and analysis of rickets in 663 children under 3 years old. Taiwan Journal of Preventive Medicine 16(4):34-35 (in Chinese)
36. Cheng CX (2010) The value of bone alkaline phosphatase in early diagnosis of rickets. Journal of Clinical Research 27(5):897-898 (in Chinese)
37. Peng LK (2010) Investigation and analysis of rickets in 980 children aged 0-3 years. Chinese Journal of School Doctor 24(9):717-720 (in Chinese)
38. Yu XL (2009) Analysis on prevalence of subclinical rickets in children aged 0-6 in three Gorges reservoir. Chinese Journal of Clinical Rational Drug Use 2(7):31-32 (in Chinese)
39. Huang XF (2008) Investigation and analysis of 664 cases of rickets in children under 3 years old. Chin Matern Child Health Care 23(9):1237-1238 (in Chinese)
40. Ning SH (2007) Application of bone alkaline phosphatase in subclinical rickets. Medical innovation research 4(6):8-9 (in Chinese)
41. Chen H (2011) Investigation report on the prevention and treatment of rickets in children aged 0-3 in Qilihe district of Lanzhou City. Chinese Journal of Healthy Birth & Child Care 17(2): 102-103 (in Chinese)
42. Guo Q (2005) Early screening and intervention of vitamin D deficiency rickets in children aged 0-3 years. Maternal and Child Health Care of China 20(23):3098-3100 (in Chinese)
43. Tang Q (2017) Investigation and prevention of rickets in 0-3 years old children. Chinese Baby 000(017):46-47 (in Chinese)
44. Ma WP (2012) Investigation and analysis of rickets about 0-3 years old children in Nanning Jiangnan district. Chinese Journal of new clinical medicine 05(008):763-765 (in Chinese)
45. Wang W, Liu L, Xue X (2014) The results of bone alkaline phosphatase (ALP) detection of 264 infants aged 0-3 years were analyzed. Contemporary Medicine Forum 000(016):35-36 (in Chinese)
46. Liu HB, Sang TG, Tian ZN (2005) Analysis of calcium deficiency in rural and city children using the BALP method. Lingnan Journal of Emergency Medicine 03:207-208 (in Chinese)
47. Pang HY, Ruan HQ, Fu SM (2004) Application of bone alkaline phosphatase in the diagnosis of rickets in children. Chinese Journal of Birth Health 12(2):112-112 (in Chinese)
48. Yang K (2008) The value of detecion of bone alkaline phosphatase during diagnosis and curative effection of vitamin D deficient rickets. Acta Medicinae Sinica 21(1):45-45 (in Chinese)
49. Guo QS, Sun GQ, Zhang SB (2002) Investigation of bone mineral density in normal people in Liaoning Province by DEXA. Chinese Journal of Osteoporosis 8(002):107-109 (in Chinese)
50. Bai MH, Ge BF, Bai J (2008) Analysis of bone mineral desity in normal people in Lanzhou area. Chinese Journal of Osteoporosis 14(010):736-737 (in Chinese)
51. Meng YJ, Wei JY, Long LY (2006) Prevalence of osteoporosis in middle-aged and elderly people in Liuzhou City. Journal of Qiqihar Medical College 27(15):1581-1581 (in Chinese)
52. Wang ZW, Ma JF, Yang DZ (2000) Investigation of bone mineral density in middle-aged and aged people in Chengdu. Chinese Journal of Osteoporosis 6(1):40-43 (in Chinese)
53. Yang CD, Yu YH (2016) Analysis of bone mineral density results of 759 healty volunteers. Journal of Harbin Medical University 50(003):261-264 (in Chinese)
54. Luo W, Liu Z, Liu HD (2020) Investigation of bone mineral density and prevalence of bone mass abnormality in 3859 healthy subjects in Beijing. Chinese Journal of Osteoporosis 26(05):101-104 (in Chinese)
55. Zhu JH, Zhang WG, Zhang YJ (2002) Determination of bone mineral density by absorptiometry in normal people in Dalian. China Journal of Orthopaedics and Traumatology 8(1):75-77 (in Chinese)
56. Zhang NN, Hao YH (2013) The investigation of bone mineral density on 1096 cases Han population in Daqing area and their incidence of osteoporosis. China Journal of Orthopaedics and Traumatology 19(10):1095-1098 (in Chinese)
57. Zhang PD, Feng YL, Zhang ZM (2008) Epidemiological investigation of bone mineral density in normal middle-aged and old people in Foshan. China Journal of Orthopaedics and Traumatology 14(8):567-569 (in Chinese)
58. Yu QY, He B, Tu M (2004) Epidemiological Investigation of Osteoprosis in Middle-aged and Aged people in Longyan Area. China Journal of Orthopaedics and Traumatology 12(3):29-31 (in Chinese)
59. Gao JH, Zheng JH, Zhang RP (2006) Bone Mineral Density Measurement and Prevalence of Osteoporosis in 2454 Citizen in Jiangmen District of Guangdong Province. Chinese General Practice 9(5):395-397 (in Chinese)
60. Luo CY, Zhang Q, Zhou CP (2009) Survey on the present status of primary osteoporosis of Hezhou district of Guangxi. Chinese Journal of Osteoporosis 15(5):357-359 (in Chinese)
61. Liu Y, Lu HR, Yu T (2014) Analysis of the Result of Bone Mineral Density in 2013 Normal People in Guangzhou Area. Modern Hospital Management 11(4):149-151 (in Chinese)
62. Zhou Ying, Pan Wei Ming, Xiao Huan (2004) Changes of bone mineral density and prevalence of osteoporosis in middle -aged and elderly people in Haikou area[J]. Chinese Journal of Osteoporosis, 2020, 26(7):1054-1058 (in Chinese)
63. Li P (2011) Study on lumbar bone mineral density and osteoporosis prevalence in middle-aged and aged people in Huhhot. Inner Mongolia Medical University (in Chinese)
64. Tan ZH, Luo WD, Li XK (2013) Analysis of the lumbar spine bone mineral density of the elderly in Huizhou region. Hainan Medical Journal 24(14):2056-2058 (in Chinese)
65. Li W (2008) Analysis of Bone Mineral Density of 411 Cases in Jinan City Proper. Shandong University (in Chinese)
66. Luo QY (2015) The investigation of related risk factors of osteoporosis and vitamin D status in women in Luzhou city. Sichuan Medical University (in Chinese)
67. Huang Y, Feng Y, Cheng Y (2016) Bone mineral density among different ethnicity in middle -aged and older people in Mianyang. Chinese Journal of Osteoporosis 22(8):1047-1049 (in Chinese)
68. Xu DB, Tu P, Wu HP (2012) Investigation on the bone mineral density in adult men in Nanchang and its elationship with smoking. Modern Preventive Medicine 39(18):4866-4869 (in Chinese)
69. Zhuo TJ, Zhou MX, Shen ZX (2002) Analysis of bone mineral density of 5186 cases by dual energy X-ray absorptiometry in Nanjing area. Chinese Journal of Osteoporosis 8(2):104-106 (in Chinese)
70. Liu F, Peng YW, Gu Z (2013) Effect of age, weight and body mass index on bone mineral density in middle-aged and aged people in YueYang city. Chinese Journal of Osteoporosis 19(9):950-953. (in Chinese)
71. Xu T, Wang XH, Luan X (2011) The analysis of bone mineral density results in 4434 healthy people in Qingdao. Chinese Journal of Osteoporosis 17(10):896-898. (in Chinese)
72. Chen ZQ, Zeng SQ, Yu LL (2011) The study of the status of bone mineral density in middle-aged and elderly adults in Xiamen City. Chinese Journal of Osteoporosis 17(10):892-896. (in Chinese)
73. Ma JL, Guo HY, Huang SX (2012) Analysis on Bone Mineral Density among Women Aged 40 to 79.9 Years in Beicai Community of Shanghai City. Journal of Community Medicine, 10(12):4-5 (in Chinese)
74. Wu FN, Zhong ZR, Han QS (2004) Investigation of peak bone mass of residents and osteoporosis morbidity on adult people in Shenzhen district. Journal of Clinical Internal Medicine 21(8):548-550 (in Chinese)
75. Zhao Y, Ye WZ, Fan YP (2002) Analysis of Bone Density Test Results in Wuxi Area. Chinese Journal of Osteoporosis 8(2):126-127 (in Chinese)
76. Jiang JS, Wang KZ, Wang CS (2009) Investigation of BMD in 1478 cases in Xi an. Chinese Journal of Osteoporosis 15(6):428-431 (in Chinese)
77. Zhang ML, Hai J (2011) Analysis of Bone Mineral Density Test Results of 1244 Cases in Xining District. Qinghai Medical Journal 41(12):63-64 (in Chinese)
78. Liu ZB, Lu H, Liu J (2012) Analysis of Bone Mineral Density Test Results of 2416 Normal People in Yanan Area. Shaanxi Medical Journal 41(6):736-738 (in Chinese)
79. Zhang XJ (2014) Research and analysis about Osteoporosis epidemiological investigation of Yizu people in Yunnan Chuxiong area and its related influencing factors. Kunming Medical University (in Chinese)
